# Glycosuria Alters Uropathogenic Escherichia coli Global Gene Expression and Virulence

**DOI:** 10.1128/msphere.00004-22

**Published:** 2022-04-28

**Authors:** Md Jahirul Islam, Kamal Bagale, Preeti P. John, Zachary Kurtz, Ritwij Kulkarni

**Affiliations:** a Department of Biology, University of Louisiana at Lafayette, Lafayette, Louisiana, USA; b Lodo Therapeutics, New York, NY, USA; University of Kentucky

**Keywords:** RNA-seq, UPEC, carbon metabolism, diabetes, glycosuria, iron utilization, urinary tract infection, uropathogenic *Escherichia coli*, virulence factors

## Abstract

Uropathogenic Escherichia coli (UPEC) is the principal etiology of more than half of urinary tract infections (UTI) in humans with diabetes mellitus. Epidemiological data and studies in mouse model of ascending UTI have elucidated various host factors responsible for increasing the susceptibility of diabetic hosts to UPEC-UTI. In contrast, diabetic urinary microenvironment-mediated alterations in UPEC physiology and its contributions to shaping UPEC-UTI pathogenesis in diabetes have not been examined. To address our central hypothesis that glycosuria directly induces urinary virulence of UPEC, we compared virulence characteristics and gene expression in human UPEC strains UTI89 (cystitis) and CFT073 (pyelonephritis), exposed for 2 h *in vitro* to urine from either male or female donors that was either plain or supplemented with glucose to mimic glycosuria. Compared to control UPEC exposed to nutrient-rich culture medium, lysogeny broth, glycosuria-exposed UPEC exhibited significant increase in biofilm formation and reduction in the hemagglutination of Guinea pig erythrocytes (a measure of type 1 piliation). In addition, the analysis of UTI89 transcriptome by RNA sequencing revealed that 2-h-long, *in vitro* exposure to glycosuria also significantly alters expression of virulence and metabolic genes central to urinary virulence of UPEC. Addition of galactose as an alternative carbon source affected biofilm formation and gene expression profile of UPEC to an extent similar to that observed with glucose exposure. In summary, our results provide novel insights into how glycosuria-mediated rapid changes in UPEC fitness may facilitate UTI pathogenesis in the diabetic urinary microenvironment.

**IMPORTANCE** Uropathogenic Escherichia coli (UPEC) is an important causative agent of urinary tract infections in diabetic humans. We examined the effects of *in vitro* exposure to glycosuria (presence of glucose in urine) on the virulence and gene expression by UPEC. Our results show that glycosuria rapidly (in 2 h) alters UPEC gene expression, induces biofilm formation, and suppresses type 1 piliation. These results offer novel insights into the pathogenesis of UPEC in the urinary tract.

## INTRODUCTION

Urinary tract infection (UTI) is a highly prevalent infection, affecting an estimated 50% of women and 20% of men at least once during their lifetimes (1, 2). Diabetes increases the risk of UTI by 1.5- to 2-fold. In 2011, an estimated 10% of total emergency department visits in the United States were by diabetic individuals seeking treatment for various infections, of which 30% of visits were specifically for urinary tract infection (UTI); in addition, an estimated 200,000 diabetic individuals required hospitalization for UTI treatment (3). The overall cost of UTI treatment is 1.2- to 1.5-fold higher for diabetic individuals than nondiabetics (4, 5). Gram-negative uropathogenic Escherichia coli (UPEC) is the principal cause of >80% of community-acquired and >40% hospital-associated UTI in humans (6). In addition, UPEC is also an important cause of more than half of all UTI cases in diabetic humans (7).

The central premise of our research is that the increased susceptibility of diabetic individuals to UTI is a function of diabetes-mediated changes in not only host immune defenses but also the virulence of microbial pathogens. Clinical studies in humans and laboratory studies using experimental induction of ascending UTI in wild-type (WT) and diabetic mice have elucidated several host factors central to the increased susceptibility of diabetic individuals to UPEC UTI: accumulation of advanced glycation end products (AGE) in diabetic urinary tract has been observed not only to be associated with bladder dysfunction but also to facilitate urinary colonization by UPEC (8–10). Diabetes-induced alterations in immune defenses such as reduction in antimicrobial peptides, proinflammatory cytokines, complement activation, and urinary leukocyte infiltration have also been shown to increase susceptibility of diabetic individuals to UTI (11–15). Given its ability to promote growth of uropathogenic bacteria, glycosuria has also been proposed to increase UTI risk in diabetics (13, 16). Whether glycosuria is a UTI risk factor has been rigorously examined in clinical trials for oral antidiabetic SGLT2i (sodium–glucose cotransporter-2 inhibitors, or gliflozins) that induce normoglycemia by preventing reuptake of glucose in the proximal convoluted tubules of nephrons, in turn increasing glucose excretion in urine (17). The results from clinical trials consistently show that even though SGLTi therapy increased UTI incidence, the UTI cases were more common at the onset of SGLT2i treatment, were of moderate severity, did not disseminate to the upper urinary tract, and were responsive to antibiotic treatment (18).

Whether glycosuria promotes bacterial virulence and whether a switch toward high-ATP yielding energy metabolism through glycolysis and the tricarboxylic acid (TCA) cycle, improves bacterial fitness in the diabetic urinary tract has not been deciphered. In this regard, we recently reported that glycosuria induces virulence mechanisms in Gram-positive Streptococcus agalactiae such as increased bacterial adherence to human bladder epithelium, resistance to antimicrobial peptide LL-37, and hemolytic ability following *in vitro* exposure to human urine supplemented with glucose (19). For the study presented here, we explored changes in UPEC physiology caused by the short-term (2-h-long) exposure to glycosuria. As model organisms, we used two human UPEC strains: UTI89, isolated from a patient with cystitis, and CFT073, isolated from a woman with pyelonephritis (20, 21). These strains were cultivated at 37°C under static conditions to induce type 1 piliation, similar to the inoculum used for the experimental induction of ascending UTI (22). We exposed type 1 piliated UPEC strains *in vitro* to nutrient-rich lysogeny broth (LB) control or plain human urine or to human urine supplemented with different glucose concentrations to mimic minimal (30 mg/dL glucose), high (300 mg/dL glucose), or severe (600 mg/dL glucose) glycosuria (16). Following 2-h-long exposure, we examined UTI89 gene expression using RNA sequencing (RNA-seq) and quantitative real-time PCR (qRT-PCR). We also compared UTI89 and CFT073 exposed to LB or urine with or without glucose for biofilm formation, hemagglutination of Guinea pig RBCs (measure of type 1 piliation), or in a mouse model of ascending UTI. UTI89 quantitative reverse transcription-PCR (qRT-PCR) and biofilm formation for UTI89 and CFT073 were also examined in urine supplemented with 600 mg/dL galactose, which is important for UPEC intracellular growth in the urinary tract (23). Our results show that glycosuria significantly alters global transcriptome of UTI89, induces virulence characteristics of UTI89 and CFT073, and strengthens our central premise that glycosuria-mediated changes in the physiology of uropathogens plays a critical role in shaping pathogenesis of UTI in diabetics. We acknowledge an important caveat that our experimental design does not examine how UPEC physiology may be affected by the long-term exposure to glycosuria either *in vitro* or inside the diabetic urinary tract.

## RESULTS

### UTI89 and CFT073 grow in human urine in the presence or absence of glucose.

**FIG 1:**
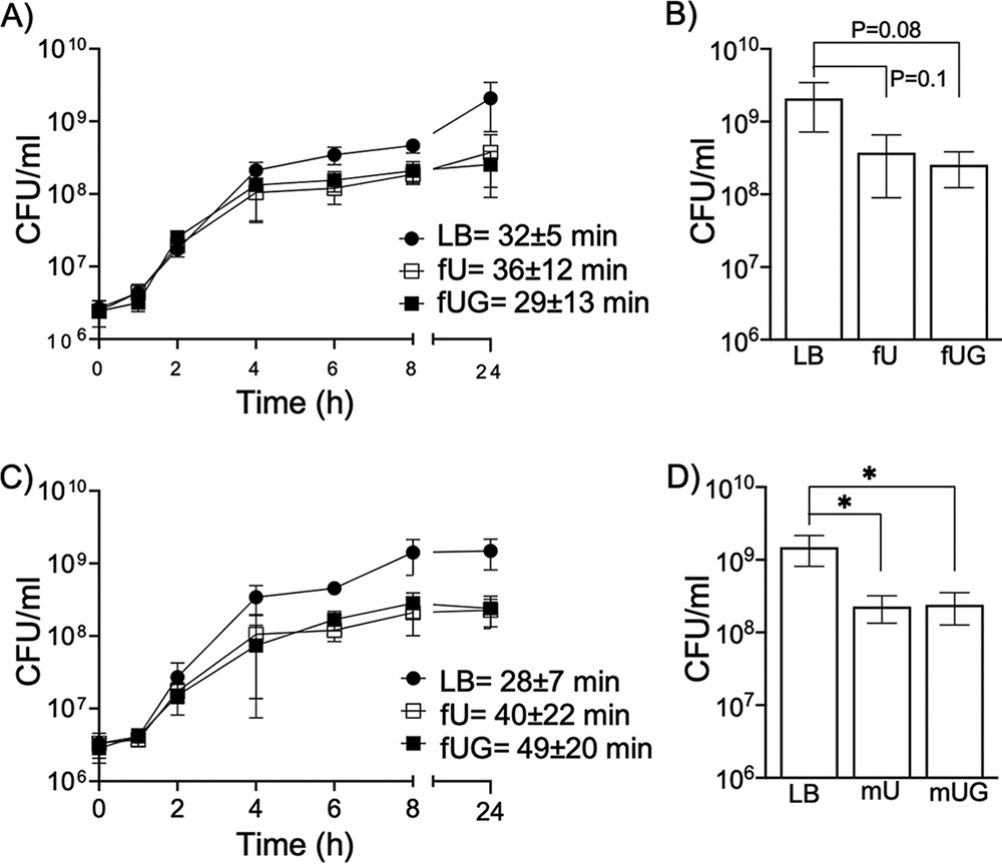
Growth of UPEC strains in the presence of LB, plain urine, or glycosuria. We monitored growth of UTI89 (A and B) or CFT073 (C and D) in LB (control), plain human female urine (fU), or in urine supplemented with 600 mg/dL glucose (fUG) by enumerating CFU/mL at various time points up to 24 h. (A and C) For every time point, average numbers of CFU/mL (three biological replicates) ± standard deviations are shown. Average doubling time in minutes ± standard deviation is shown next to the plot for each specific exposure. (B and D) Numbers of CFU/mL at 24 h from 3 biological replicates were compared using unpaired, two-tailed *t* test (where *P* value is not shown, * represents *P *≤ 0.05).

To confirm that UPEC survives in human urine either in the presence or absence of glucose, we cultivated type 1 piliated UTI89 and CFT073 in LB, female urine alone (fU), or fU plus 600 mg/dL glucose (fUG) at 37°C for 24 h under static conditions. CFU were enumerated at 0, 1, 2, 4, 6, 8, and 24 h. Using ordinary one-way analysis of variance (ANOVA) followed by Tukey’s multiple comparisons, we did not observe statistically significant change in the doubling times (DT) of UTI89-LB, UTI89-fU, UTI89-fUG, CFT073-LB, CFT073-fU, or CFT073-fUG (Fig. 1A and C). These results suggested that the presence of glucosuria does not directly increase UPEC growth rate. Compared to their respective LB controls, both UTI89 and CFT073 cultivated in either fU or fUG showed 5- to 8-fold reduction in CFU/mL at 24-h time point (Fig. 1B and D).

### RNA-seq and read mapping.

We used transcriptome sequencing (RNA-seq) to quantify differential gene expression by assessing variation across the transcriptomes of UTI89-LB (control), UTI89-fU, and UTI89-fUG. RNA was isolated from three independent biological replicates for each treatment. The raw Illumina reads are available at NCBI BioProject database accession no. PRJNA786257. UTI89 was selected for RNA-seq as it is a well-studied bladder isolate from acute cystitis patient (20). Whole-transcriptome sequencing with rRNA depletion resulted in an average of 23 million 150-bp paired-end reads per sample (range, 14 to 37.4 million reads). After adapter trimming and quality filtering, we retained an average of 96.5% of reads (94.7 to 98.6%) per library. Subsequently, an average of 21.7 million reads (13.4 to 34.7 million reads) per library was successfully mapped to Escherichia coli UTI89 (O8:K1:H7) reference genome (GenBank accession no. CP000243). Euclidean distances between samples and principal-component analysis for comparisons between UTI89-fU and UTI89-LB (see Fig. S1A and B in the supplemental material), UTI89-fUG and UTI89-LB (Fig. S1C and D), and UTI89-fUG and UTI89-fU (Fig. S1E and F) revealed that expression patterns in biological replicates of each treatment group were more similar to each other than they were to those in biological replicates of the contrasting treatment group. Detailed RNA-seq data (normalized counts for triplicate samples), log_2_FC (fold change), and *P*adj (adjusted *P* value) for all genes are presented in Data Set S1, Tab 1 (UTI89-LB versus UTI89-fU), Tab 2 (UTI89-fUG versus UTI89-LB), and Tab 3 (UTI89-fUG versus UTI89-fU). In UTI89-LB versus UTI89-fU comparison, expression of 638 genes was significantly altered (defined as absolute log_2_FC of >1 and *P*adj of ≤0.05). Of these, 393 were significantly upregulated and 245 significantly downregulated. In UTI89-LB versus UTI89-fUG comparison, of 846 differentially expressed genes, 395 were significantly upregulated and 245 significantly downregulated. In UTI89-fUG versus UTI89-fU comparison, of 691 differentially expressed genes, 253 were significantly upregulated and 438 significantly downregulated. RNA-seq results were confirmed by qRT-PCR. Fold change for UTI89-LB versus UTI89-fU and UTI89-LB versus UTI89-fUG was correlated across the genes, with *r*^2^ = 0.83 and *r*^2^ = 0.93, respectively (Fig. S2).

10.1128/msphere.00004-22.1 FIG S1 Principal-component analysis and Euclidean distance between samples for UTI89-LB versus UTI89-fU (A and B), UTI89-LB versus UTI89-fUG (C and D), and UTI89-fU versus UTI89-fUG (E and F). Download FIG S1, PDF file, 0.6 MB. Copyright © 2022 Islam et al. 2022 Islam et al. https://creativecommons.org/licenses/by/4.0/ This content is distributed under the terms of the Creative Commons Attribution 4.0 International license.

10.1128/msphere.00004-22.2 FIG S2 Correlation between qRT-PCR and RNA-seq results for UTI89-fU compared to UTI89-LB (A) and UTI-89-fUG compared to UTI89-LB (B). Download FIG S2, PDF file, 0.04 MB. Copyright © 2022 Islam et al. 2022 Islam et al. https://creativecommons.org/licenses/by/4.0/ This content is distributed under the terms of the Creative Commons Attribution 4.0 International license.

10.1128/msphere.00004-22.6 DATA SET S1 Detailed RNA-seq data for all genes. Shown are KEGG ID, gene symbols, normalized counts for each biological replicate, log_2_(FC), *P*adj, and GenBank annotation data for UTI89-LB versus UTI89-fU (Tab 1), UTI89-fUG versus UTI89-LB (Tab 2), and UTI89-fUG versus UTI89-fU (Tab 3) comparisons. Download Data Set S1, XLSX file, 1.9 MB. Copyright © 2022 Islam et al. 2022 Islam et al. https://creativecommons.org/licenses/by/4.0/ This content is distributed under the terms of the Creative Commons Attribution 4.0 International license.

### Glycosuria affects expression of UTI89 genes involved in urinary fitness.

The major objective of RNA-seq analysis was to define changes in UTI89 gene expression caused by glycosuria. Hence, this section primarily describes changes in the gene expression in UTI89-fUG compared to UTI89-fU. The data are summarized in tables showing differential gene expression data where |log_2_FC| is <1 and *P*adj is <0.05 for UTI89-fU versus UTI89-fUG comparison. Differential gene expression data for UTI89-fU versus UTI89-LB and UTI89fUG versus UTI89-LB comparisons can be found in supplemental tables.

### (i) UTI pathogenesis.

We focused on the expression of genes encoding UPEC virulence factors, such as adhesins, toxins, and virulence regulators, and genes from KEGG pathway *eci02026* biofilm formation, shown in Table 1, while genes under KEGG category human diseases are in Table 2. Biofilm formation in E. coli is regulated primarily via secondary messenger cAMP (3′,5′ cyclic AMP)-CRP (cAMP receptor protein) complex, which induces biosynthesis of surface adhesive type 1 pili, curli fibers, flagella, and K1 capsule and suppresses sigma factor *RpoS*, a known repressor of initiation of biofilms (24, 25). Glucose-mediated catabolite repression suppresses E. coli biofilms by downregulation of *cyaA*, encoding enzyme adenylyl cyclase, which catalyzes cAMP production (24). Compared to UTI89-fU, UTI89-fUG showed significantly reduced expression of the regulators of curli fiber (*csgD*, UTI89_C1161) and flagellar biosynthesis (*flhCD*, UTI89_C2094/C2094) and of *kpsF* (UTI89_C3362), encoding K1 capsule. These observations suggest that glycosuria may augment UPEC biofilms (shown in Fig. 4) through molecular effectors other than type 1 pili, curli fibers, flagella, or K1 capsule. These observations must be confirmed in the future by examining the effects of glycosuria on biofilm formation by UPEC mutants ablated in one or more of these adhesins.

**TABLE 1:** RNA-seq results for virulence genes^*a*^

KEGG ID	Gene name	Level (log_2_FC) for^*b*^:			GenBank definition
		LB vs fU	LB vs fUG	fU vs fUG	
UTI89_C0149		1.0177	−0.0745	−1.0715*	Fimbria-like adhesin
UTI89_C0152		0.7837	−0.9048	−1.6806*	Fimbrial protein
UTI89_C1159	*csgF*	1.1020	−1.2543*	−2.3179*	Curli production assembly/transport protein CsgF
UTI89_C1160	*csgE*	0.9886	−1.2301	−2.2080*	Curli production assembly/transport protein CsgE
UTI89_C1161	*csgD*	1.1839	−0.8703	−2.0304*	Transcriptional regulator CsgD
UTI89_C2094	*flhC*	0.6029	−1.1429	−1.7342*	Flagellar transcriptional regulator FlhC
UTI89_C2095	*flhD*	0.7667	−1.4090*	−2.1631*	Flagellar transcriptional regulator FlhD
UTI89_C2151	*rcsA*	−0.1543	−1.1939*	−1.0163*	Transcriptional regulator RcsA
UTI89_C3049	*luxS*	−0.3816	0.6139	1.0096*	*S*-Ribosylhomocysteine lyase
UTI89_C3935		−0.0955	−1.1975*	−1.0802*	Fimbria/pilus periplasmic chaperone
UTI89_C4639	*kguS*	0.4967	−1.0320*	−1.4958*	Sensor histidine kinase
d-Serine and l-serine metabolism					
UTI89_C2010	*sdaA*	−0.1862	−1.4675*	−1.4675*	l-Serine ammonia-lyase
UTI89_C3168	*sdaB*	−0.6305	−2.2129*	−2.2129*	l-Serine ammonia-lyase II
UTI89_C3167	*sdaC*	−0.2304	−2.8554*	−2.8554*	HAAAP family serine/threonine permease SdaC

a Includes genes from KEGG pathway biofilm formation (*eci02026*) and genes involved in metabolism and transport of d-serine (*dsdAXC*) and l-serine (*sdaABC*), shown to play a role in the uropathogenesis.

b Only values with absolute log_2_FC > 1 and *P*adj < 0.05 (denoted by an asterisk) in UTI89-fU versus UTI89-fUG comparison are shown.

**TABLE 2:** RNA-seq results for genes from human diseases category in KEGG database

KEGG ID	Gene name	Level (log_2_FC) for^*a*^:			GenBank definition
		LB vs fU	LB vs fUG	fU vs fUG	
UTI89_C0095	*murF*	−0.7417	0.3595	1.1134*	UDP-*N*-acetylmuramoyl-tripeptide—d-alanyl-d-alanine ligase
UTI89_C0096	*mraY*	−0.9774	0.1922	1.1833*	Phospho-*N*-acetylmuramoyl-pentapeptide-transferase
UTI89_C0099	*murG*	−1.2970*	−0.0013	1.3099*	Undecaprenyldiphospho-muramoylpentapeptide beta-*N*-acetylglucosaminyltransferase
UTI89_C0275		0.1184	−1.0975*	−1.1988*	RNA ligase RtcB family protein
UTI89_C0626	*pagP*	−0.8325	0.3986	1.2467*	Lipid IV(A) palmitoyltransferase PagP
UTI89_C1001	*ompF*	0.1189	−2.1144*	−2.2156*	Porin OmpF
UTI89_C1376	*dadX*	0.0759	−1.5158*	−1.5720*	Catabolic alanine racemase DadX
UTI89_C1601	*mppA*	0.1456	−1.3388*	−1.4675*	Murein tripeptide ABC transporter substrate-binding protein MppA
UTI89_C1750	*marA*	3.2992*	1.7309*	−1.5534*	MDR efflux pump AcrAB transcriptional activator MarA

a Only values of absolute log_2_FC > 1 and *P*adj < 0.05 (denoted by an asterisk) in UTI89-fU versus UTI89-fUG comparison are shown.

Table 2 shows differential expression of genes categorized under human diseases, which includes KEGG pathways for *eci01501-*beta-lactam resistance, *eci01502-*vancomycin resistance, and *eci01503-*cationic antimicrobial peptide (CAMP) resistance. Compared to UTI89-fU, UTI89-fUG showed significantly increased expression of *murF* (UTI89_C0095), *mraY* (UTI89_C0096), *murG* (UTI89_C0099), and *murC* (UTI89_C0101) and CAMP resistance gene *pagP* (UTI89_C0626). Mur enzymes (MurABCDEF) catalyze synthesis of UDP *N*-acetyl muramyl pentapeptide from UDP *N-*acetylglucosamine, a major component of UPEC cell wall peptidoglycan, which determines bacterial shape and mediates protection. Of these, *murCDEF* were significantly upregulated in UTI89-fUG compared to UTI89-fU. In contrast, β-lactam resistance genes *ampG* (UTI89_C0275, UTI89_C0457), *ompF* (UTI89_C1001), and *mppA* (UTI89_C1601) were significantly downregulated in UTI89-fUG compared to UTI-fU.

### (ii) Energy metabolism.

**FIG 2:**
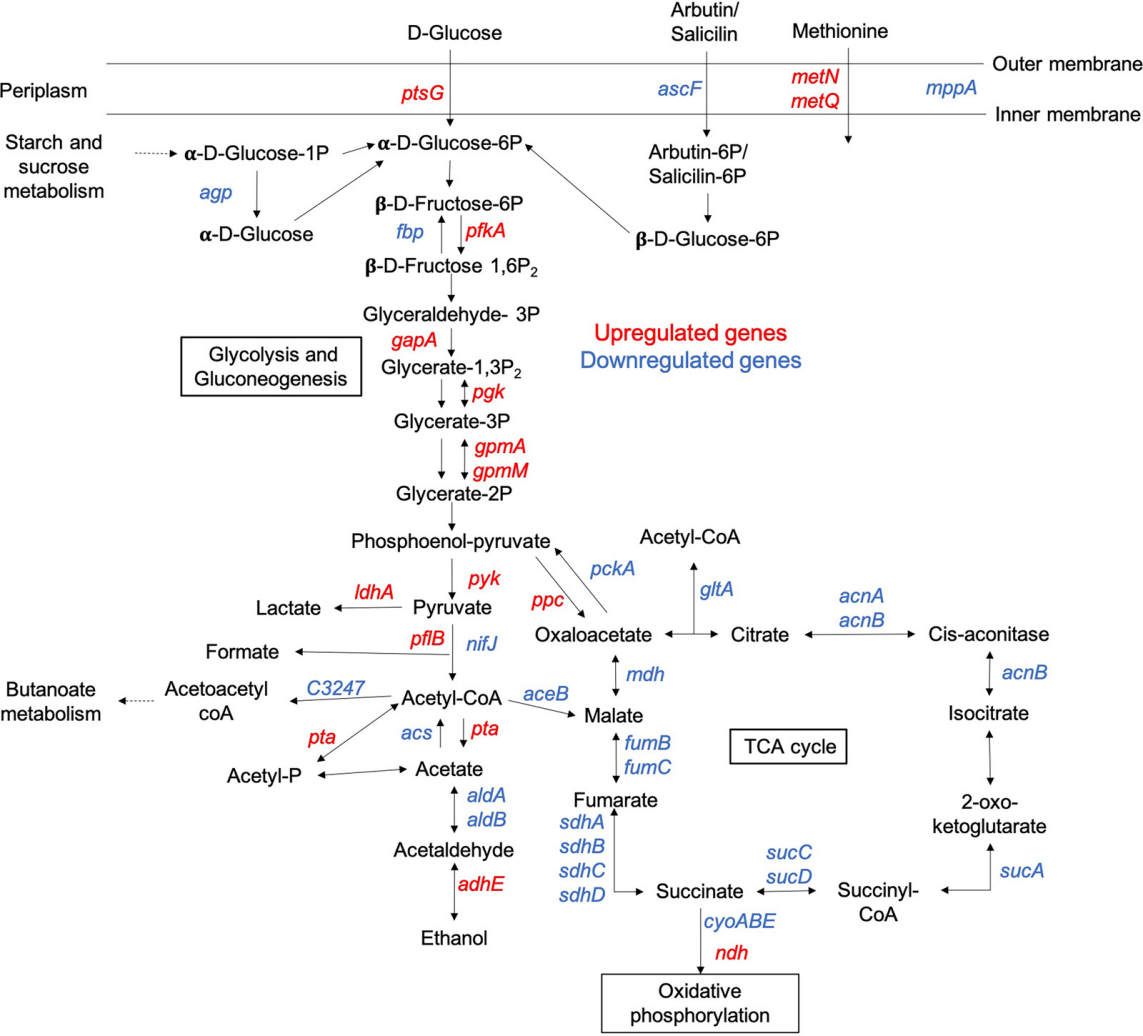
Effects of glycosuria on the expression of genes encoding enzymes from central carbon metabolism pathways. Differential gene expression (where |log_2_FC| ≥ 1 and *P*adj ≤ 0.05) for UTI89-fUG versus UTI89-fU comparison is shown in the context of steps in glycolysis/gluconeogenesis, pyruvate oxidation, and TCA cycle. The schematic is adapted from KEGG; significantly upregulated genes are shown in red, and significantly downregulated genes are shown in blue. See Table 3 for more details.

The lower gastrointestinal tract is the primary and immediate reservoir for UPEC before it gains access to the urinary tract via fecal contamination of peri-urethral area and causes ascending UTI (26, 27). When transitioning from nutritionally rich gastrointestinal microenvironment to a nondiabetic urinary tract without glycosuria, UPEC switches to catabolizing amino acids and short peptides present at low concentration in urine (28, 29). For this, amino acids are transported across UPEC membrane and catabolized via TCA cycle and gluconeogenesis to synthesize glucose (28, 29). We observed that in the presence of glycosuria, central carbon metabolism was primarily glycolytic, as evidenced by significant enrichment of KEGG pathways for glycolysis/gluconeogenesis, pyruvate metabolism, and TCA cycle in UTI89-fUG versus UTI89-fU comparison (Fig. S3C). Significantly upregulated and downregulated genes from UTI89-fUG versus UTI89-fU comparison are also presented in the context of central carbon metabolism pathways in Fig. 2.

10.1128/msphere.00004-22.3 FIG S3 Enrichment analysis for RNA-seq data. KEGG pathways significantly enriched for DEG at *P *< 0.01 are shown for three comparisons. (A) UTI89-LB versus UTI89-fU. (B) UTI89-fUG versus UTI89-LB. (C) UTI89-fUG versus UTI89-fU. For each KEGG pathway, the number of DEG is shown next to the histogram. Download FIG S3, PDF file, 0.3 MB. Copyright © 2022 Islam et al. 2022 Islam et al. https://creativecommons.org/licenses/by/4.0/ This content is distributed under the terms of the Creative Commons Attribution 4.0 International license.

As expected, compared to UTI89-LB, both UTI89-fU and UTI89-fUG showed upregulation of genes encoding transporters of amino acids serine (*dsdX*, UTI89_C4956; *cycA*, UTI89_C4817; *sstT*, UTI89_C3527), arginine (*artJMQJP*, UTI89_C0863-67), histidine (*hisQJ*, UTI89_C2592/93), and branched-chain amino acids (*livFGMHKJ*, UTI89_C3961-65, C3969) and dipeptides (*dppBA*, UTI89_C4081/82). This corresponds to the metabolic switch toward amino acid catabolism in glucose-limiting human urine. In contrast, UTI89-fUG showed significant upregulation of methionine ABC transporter genes *metQN* (UTI89_C0213/0215) compared to UTI89-fU (Table 3).

**TABLE 3:** RNA-seq analysis of expression of genes involved in energy metabolism

KEGG ID	Gene name	Level (log_2_FC) for^*a*^:			GenBank definition
		LB vs fU	LB vs fUG	fU vs fUG	
Amino acid and peptide transport					
UTI89_C0213	*metQ*	−0.5782	2.9934*	3.5851*	Methionine ABC transporter substrate-binding lipoprotein MetQ
UTI89_C0215	*metN*	−0.6530	3.5251*	4.1934*	Methionine ABC transporter ATP-binding protein MetN
UTI89_C1601	*mppA*	0.1456	−1.3388*	−1.4674*	Murein tripeptide ABC transporter substrate-binding protein MppA
Glycolysis and gluconeogenesis					
UTI89_C0752	*gpmA*	0.0101	2.0387*	2.0473*	2,3-Diphosphoglycerate-dependent phosphoglycerate mutase
UTI89_C1065	*agp*	−0.1258	−1.7691*	−1.6250*	Bifunctional glucose-1-phosphatase/inositol phosphatase
UTI89_C1228	*ptsG*	0.0854	1.3923*	1.3224*	PTS glucose transporter subunit IIBC
UTI89_C1975	*gapA*	−0.0356	1.7083*	1.7599*	Glyceraldehyde-3-phosphate dehydrogenase
UTI89_C2058	*pyk*	−0.5710	0.4195	1.0021*	Pyruvate kinase
UTI89_C3077	*ascF*	1.2493*	−0.0906	−1.3217*	PTS cellobiose/arbutin/salicin transporter subunit IIBC
UTI89_C3309	*pgk*	−0.3316	0.6584	1.0055*	Phosphoglycerate kinase
UTI89_C3903	*pckA*	−0.3553	−2.8086*	−2.4364*	Phosphoenolpyruvate carboxykinase (ATP)
UTI89_C4129	*aldB*	0.6100	−2.1970*	−2.7870*	Aldehyde dehydrogenase AldB
UTI89_C4157	*gpmM*	−0.2299	1.5524*	1.7966*	2,3-Bisphosphoglycerate-independent phosphoglycerate mutase
UTI89_C4499	*pfkA*	−0.0932	1.6914*	1.7999*	6-Phosphofructokinase
UTI89_C4836	*Fbp*	0.0150	−1.1007*	−1.0980*	Class 1 fructose-bisphosphatase
Pyruvate metabolism					
UTI89_C0974	*pflB*	−0.7838	1.6661*	2.4615*	Formate C-acetyltransferase
UTI89_C1623	*ldhA*	0.4059	2.1198*	1.7317*	d-Lactate dehydrogenase
UTI89_C1637	*aldA*	1.0333	−1.7415*	−2.7567*	Aldehyde dehydrogenase
UTI89_C2581	*pta*	−0.4943	0.7184	1.2222*	Phosphate acetyltransferase
UTI89_C3247		0.5758	−2.5276*	−3.0862*	Acetyl-CoA C-acetyltransferase
UTI89_C4547	*ppc*	0.3527	1.7797*	1.4436*	Phosphoenolpyruvate carboxylase
UTI89_C4573	*aceB*	2.3951*	−0.6913	−3.0656*	Malate synthase A
TCA cycle					
UTI89_C0131	*acnB*	−0.0358	−1.5379*	−1.4844*	Bifunctional aconitate hydratase 2/2-methylisocitrate dehydratase
UTI89_C0715	*gltA*	0.5582	−1.3136*	−1.8526*	Citrate (Si)-synthase
UTI89_C0717	*sdhC*	0.8090	−4.4294*	−5.2186*	Succinate dehydrogenase cytochrome b556 subunit
UTI89_C0718	*sdhD*	0.7750	−4.8307*	−5.5868*	Succinate dehydrogenase membrane anchor subunit
UTI89_C0719	*sdhA*	0.5266	−4.2492*	−4.7579*	Succinate dehydrogenase flavoprotein subunit
UTI89_C0720	*sdhB*	0.2258	−2.5564*	−2.7659*	Succinate dehydrogenase iron-sulfur subunit SdhB
UTI89_C0721	*sucA*	0.0126	−2.0687*	−2.0640*	2-Oxoglutarate dehydrogenase E1 component
UTI89_C0723	*sucC*	0.0118	−1.5590*	−1.5540*	ADP-forming succinate—CoA ligase subunit beta
UTI89_C0724	*sucD*	0.0688	−1.5421*	−1.5935*	Succinate-CoA ligase subunit alpha
UTI89_C1438	*adhE*	−0.0074	2.0939*	2.1154*	Bifunctional acetaldehyde-CoA/alcohol dehydrogenase
UTI89_C1547	*acnA*	0.0207	−1.8463*	−1.8493*	Aconitate hydratase AcnA
UTI89_C1619	*nifJ* ^*b*^	0.6094	−0.5765	−1.1706*	Pyruvate:ferredoxin (flavodoxin) oxidoreductase
UTI89_C1799	*fumC*	2.1357*	−0.6150	−2.7316*	Class II fumarate hydratase
UTI89_C1800	*fumB*	0.1454	−3.0780*	−3.2048*	Class I fumarate hydratase
UTI89_C3667	*mdh*	1.1211*	−0.2544	−1.3580*	Malate dehydrogenase
UTI89_C4659	*acs*	1.1794	−1.7217*	−2.8835*	Acetate-CoA ligase
Electron transport chain					
UTI89_C0455	*cyoB*	1.026	−1.855*	−2.863*	Cytochrome *o* ubiquinol oxidase subunit I
UTI89_C0456	*cyoA*	1.095	−2.273*	−3.347*	Cytochrome *o* ubiquinol oxidase subunit II
UTI89_C0451	*cyoE*	0.473	−0.940	−1.398*	Protoheme IX farnesyltransferase
UTI89_C1237	*ndh*	−1.591*	−0.072	1.536*	NADH-quinone dehydrogenase

a Only values with absolute log_2_FC > 1 and *P*adj < 0.05 (denoted by an asterisk) in UTI89-fU versus UTI89-fUG comparison are shown.

b *nifJ* encodes enzyme catalyzing pyruvate oxidation.

Compared to UTI89-fU, UTI89-fUG showed significant upregulation of glycolysis genes *gpmA* (UTI89_C0752), *gapA* (UTI89_C1975), *pfkA* (UTI89_C2058), *pgk* (UTI89_C3309), *gpmM* (UTI89_C4157), and *pyk* (UTI89_C4499). UTI89-fUG also showed significant downregulation of gluconeogenesis genes *pckA* (UTI89_C3903, pyruvate carboxykinase) and *fbp* (UTI89_4836, fructose 1,6 bisphosphatase), encoding enzymes that catalyze bypass of irreversible steps of glycolysis, and of *fumB* (UTI89_C1800, fumarate hydratase), catalyzing conversion of malate to fumarate in reverse TCA cycle. Compared to UTI89-fU, UTI-fUG also exhibited significant downregulation of α-ketoglutarate utilization genes *sucACD* (UTI89_C0721-23), which may be attributed to significantly reduced expression of *kguS* (UTI89_C4639) sensor kinase in UTI89-fUG compared to UTI89-fU, as shown in Table 1. Previous work has shown that *kguSR* two-component system facilitates UPEC pathogenesis in the urinary tract via regulating the expression of proteins involved in the transport of α-ketoglutarate and its utilization as a sole source of carbon (30). Overall, these results indicate that exposure to glycosuria induces metabolic transition of UPEC to glycolysis. In contrast, UTI89-fU exhibited significant upregulation of *fumC* (UTI89_1799, fumarate hydratase catalyzes fumarate oxidation to malate) and *mdh* (UTI89_ 3667, malate dehydrogenase catalyzes malate to oxaloacetate), while expression of gluconeogenesis gene *pckA*, *fbp*, or *fumB* was not significantly altered in UTI89-fU. Serine, present in large amounts in urine, is preferentially catabolized by UPEC into acetate (29, 31). For the genes encoding enzymes involved in serine-to-acetate catabolism, we observed significant downregulation of phosphate acetyltransferase (*pta*, UTI89_C2782) in UTI89-fUG, while acetate kinase (*ackA*, UTI89_C2579) was unchanged in either UTI89-fU or UTI89-fUG. UTI89-fUG also showed significant downregulation of acetate-coenzyme A (CoA) ligase (*acs*, UTI89_C4659), involved in acetate utilization (32).

### (iii) Metal ion transporters.

In response to iron-limiting urinary microenvironment, UPEC upregulates iron acquisition system genes (33). Hence, we examined RNA-seq data for differential expression of genes encoding metal ion transporters (iron, manganese, nickel, cobalt, copper, and zinc), which constitute important determinants of urinary fitness and virulence of UPEC (Table 4). Exposure to glycosuria (UTI89-fUG) showed significant upregulation of iron uptake systems importing siderophores, such as yersiniabactin (*ybt*-*fyuA*, UTI89_C2178-C2188), enterobactin (*fepAECB*, UTI89_C0584/89/90/94), and salmochelin (*iroCB*, UTI89_C1121/22), free ferrous/manganese ion importer *sitDCBA* (UTI89_C1336-39), and *chuATY* (UTI89_C4028/4033/4036), importer of iron bound to host protein heme. We did not observe changes in the expression of genes encoding transporter systems for copper (*copAD* and *cusSRCBA*), nickel (*nikBCADER*), or zinc (*znuCBA* and *zntBRA*) in UTI89-fUG compared to UTI89-fU.

**TABLE 4:** RNA-seq analysis of expression of genes involved in metal transport

KEGG ID	Gene name	Level (log_2_FC) for^*a*^:			GenBank definition
		LB vs fU	LB vs fUG	fU vs fUG	
UTI89_C1121	*iroC*	0.5412	1.5493*	1.0244*	ABC transporter ATP-binding protein
UTI89_C1122	*iroB*	1.2711	3.1580*	1.9088*	Glycosyltransferase
UTI89_C1336	*sitD*	1.376*	3.355*	1.997*	Iron/manganese ABC transporter permease subunit SitD
UTI89_C1337	*sitC*	0.959	2.552*	1.612*	Iron/manganese ABC transporter permease subunit SitC
UTI89_C1338	*sitB*	0.816	2.338*	1.543*	Iron/manganese ABC transporter ATP-binding protein SitB
UTI89_C1339	*sitA*	0.960	2.363*	1.423*	Iron/manganese ABC transporter substrate-binding protein SitA
UTI89_C2178	*ybtS*	1.8829*	4.6733*	2.8072*	Yersiniabactin biosynthesis salicylate synthase YbtS
UTI89_C2179	*ybtX*	1.8301*	4.2234*	2.4069*	Yersiniabactin-associated zinc MFS transporter YbtX
UTI89_C2180	*ybtQ*	1.5576*	3.9076*	2.3676*	Yersiniabactin ABC transporter ATP-binding/permease protein YbtQ
UTI89_C2181	*ybtP*	2.2513*	4.5383*	2.3051*	Yersiniabactin ABC transporter ATP-binding/permease protein YbtP
UTI89_C2182	*ybtA*	0.0385	1.4485*	1.4319*	Yersiniabactin transcriptional regulator YbtA
UTI89_C2183	*irp2*	2.5318*	4.9924*	2.4791*	Yersiniabactin nonribosomal peptide synthetase HMWP2
UTI89_C2184	*irp1*	0.6635	3.0529*	2.4055*	Yersiniabactin polyketide synthase HMWP1
UTI89_C2185	*ybtU*	1.5129*	4.2077*	2.7156*	Yersiniabactin biosynthesis oxidoreductase YbtU
UTI89_C2186	*ybtT*	2.9422*	5.8193*	2.8999*	Yersiniabactin biosynthesis thioesterase YbtT
UTI89_C2187	*ybtE*	1.4342*	3.9199*	2.5027*	Yersiniabactin biosynthesis salycil-AMP ligase YbtE
UTI89_C2188	*fyuA*	2.6006*	4.0152*	1.4343*	Siderophore yersiniabactin receptor FyuA
UTI89_C4033	*chuT*	2.2713*	3.8607*	1.6114*	Hemin ABC transporter substrate-binding protein
UTI89_C4036	*chuY*	2.0210*	3.7934*	1.7908*	Anaerobilin reductase

a Only values with absolute log_2_FC > 1 and *P*adj < 0.05 (denoted by an asterisk) in UTI89-fU versus UTI89-fUG comparison are shown.

### qRT-PCR analysis of UPEC gene expression.

**FIG 3:**
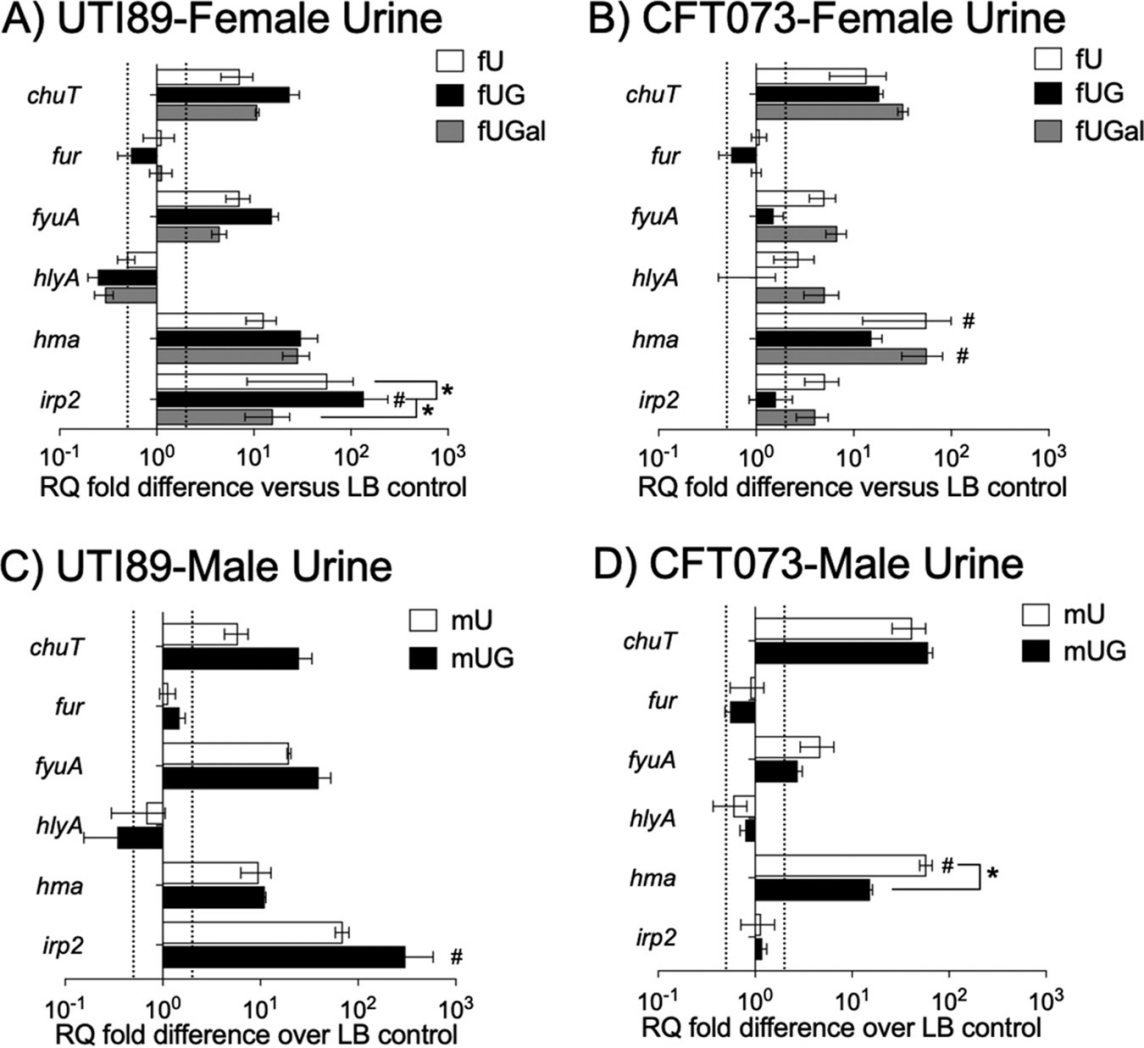
qRT-PCR results for UPEC strains exposed to urine plain or supplemented with glucose or galactose. We used quantitative real-time PCR (qRT-PCR) to determine mRNA transcript levels for specific virulence and associated genes (indicated on *y* axis) with normalization to 16S rRNA in UTI89 (A and C) or CFT073 (B and D) following 2-h-long exposure to fU, fUG, or fUGal (A and B) or to mU or mUG (C and D). RQ values were calculated by comparative threshold cycle (ΔΔ*C_T_*) algorithm. RQ fold differences over transcript levels from LB-control are presented as averages from at least two biological replicates (each with three technical repeats) ± standard deviations. Dotted lines indicate 2-fold up- or downregulation. #, *P *< 0.05 compared to UTI89-LB or CFT073-LB; *, *P* < 0.05 for other comparisons as shown. Nonsignificant *P* values are not shown.

Using qRT-PCR, we examined mRNA transcript levels in UTI89-LB, UTI89-fU, and UTI89-fUG for a panel of 23 genes involved in UPEC virulence, metabolism, and metal ion transport (Table 5 and Fig. S4). Comparisons UTI-fU versus UTI89-LB and UTI89-fUG versus UTI89-LB showed significant correlation with RNA-seq results across the panel of 23 genes (Fig. S2). From this panel, we chose six genes, namely, *hlyA* (UTI89_C4926), *chuT* (UTI89_C4033), *fur* (UTI89_C0687), *fyuA* (UTI89_C2188), *hma* (UTI89_C2234), and *irp2* (UTI89_C2183), for further examination of gene expression in UTI89 (Fig. 3A and C) or CFT073 (Fig. 3B and D) following exposure to LB, fU, fUG, or female urine plus 600mg/dL galactose (fUGal) (Fig. 3A and B) or to LB, plain male urine (mU), or male urine plus 600 mg/dL glucose (mUG) (Fig. 3C and D). Transcript levels for specific genes were determined using qRT-PCR with normalization to 16S rRNA. Relative quantification was then calculated using comparative threshold cycle (ΔΔ*C_T_*) (34) followed by the determination of fold difference over transcript levels in UPEC exposed to LB. We observed that gene expression profiles for UTI89-fUGal and CFT073-fUGal were similar to those observed in UTI89-fUG and CFT073-fUG, respectively (Fig. 3A and B). In addition, gene expression profiles were similar irrespective of whether the UPEC strains were exposed to the urine from male or female donors (Fig. 3A and C for UTI89 and Fig. 3B and D for CFT073). Of note, *irp2* expression was upregulated in UTI89 but not in CFT073 following exposure to either male or female urine with or without glucose.

**TABLE 5:** Primers used in this study

Primer name	Sequence
16S FOR	GGC GCA TAC AAA GAG AAG
16S REV	ATG GAG TCG AGT TGC AGA
*ackA* FOR	AAT GTT TCC ACC TGC CCG AA
*ackA* REV	AAA GTT GAG CGC TTC GCT GTG A
*acs* FOR	TAG CTA TAA AGA GCT GCA CCG CGA
*acs* REV	GCT TCC GGT ACC ATC GGC ATA TAA
*chuT* FOR	GAT TGC GGC TAA CCC TGA AG
*chuT* REV	TCA ACG GTG ATA ATG CGC TG
*cnf1* FOR	GGC GAC AAA TGC AGT ATT GCT TGG
*cnf1* REV	GAC GTT GGT TGC GGT AAT TTT GGG
*csgA* FOR	GTA GCA GCA ATT GCA GCA ATC G
*csgA* REV	TTA GAT GCA GTC TGG TCA ACA G
*cusC* FOR	AAT GTC GCG CAA AGC TAT TT
*cusC* REV	CGA CAA ACG CAT ATG ACT GC
*fdhF* FOR	GAG CGC CAT CAA AGA GAA GT
*fdhF* REV	GCG CAA ATT TTT GCA TTA CA
*fimA* FOR	ACT CTG GCA ATC GTT GTT CTG TCG
*fimA* REV	ATC AAC AGA GCC TGC ATC AAC TGC
*fimH* FOR	CAA TGG TAC CGC AAT CCC TA
*fimH* REV	GCA GGC GCA AGG TTT ACA
*fliC* FOR	ACA GCC TCT CGC TGA TCA CTC AAA
*fliC* REV	GCG CTG TTA ATA CGC AAG CCA GAA
*frr* FOR	CTT GTT CTG CTT CAC CAC GA
*frr* REV	GGC AAG CGT AAC GGT AGA AG
*fur* FOR	TCT GGC TAC GGT ATA TCG CG
*fur* REV	CGT GGT GAT GTT GCT GTG TT
*fyuA* FOR	GGT CTT GAT GCC AAA CCG TT
*fyuA* REV	GGT ATA AAA CGT CGC GGC TT
*gdhA* FOR	TCC TCG GCT TCG AAC AAA CCT TCA
*gdhA* REV	GGC AGA AAC GCA TAA CTT CGC CTT
*glnA* FOR	TTT GCG CTT CAC CGA TAC CAA AGG
*glnA* REV	TTA ATG CCT TTC CAG CCG CCA ATC
*hlyA* FOR	GCA AAT AAA TTG CAC TCA GCA G
*hlyA* REV	CAA GGT CAT TAA GGC TTG AAC C
*hma* FOR	ATC GTT CGG CAA GCA ACC TTT G
*hma* REV	ATG CGG ATT TGT TTA CGG CCT G
*irp2* FOR	AAG GAT TCG CTG TTA CCG GAC
*irp2* REV	AAC TCC TGA TAC AGG TGG C
*kdpA* FOR	CTC TGA CCG CGA GAT GAA CT
*kdpA* REV	GTG CTG ACC GAG CAA CAT AA
*nikC* FOR	GCT GTA TCG CGA CAT TCT GA
*nikC* REV	TTA CCC AGT TCC GGT TTT GA
*papC* FOR	GTG GCA GTA TGA GTA ATG ACC GTT A
*papC* REV	ATA TCC TTT CTG CAG GGA TGC AAT A
*sfa* FOR	TGG CCA CCG GTC TTA TTA AC
*sfa* REV	CCCTCCCTGTAACAGTAATCGT
*trkA* FOR	GTG CGC GAT GCC GAT AAG CTA TTT
*trkA* REV	TCA GCG AAG TTC ACC ACC TGC AAT
*usp* FOR	ACA TTC ACG GCA AGC CTC AG
*usp* REV	AGC GAG TTC CTG GTG AAA GC

10.1128/msphere.00004-22.4 FIG S4 qRT-PCR analysis of expression of metabolic and virulence genes. RQ fold differences for specific mRNA transcript levels for UTI89-fU or UTI89-fUG over transcript levels from LB-control are presented as averages from at least two biological replicates (each with three technical repeats) ± standard deviations. Download FIG S4, PDF file, 0.04 MB. Copyright © 2022 Islam et al. 2022 Islam et al. https://creativecommons.org/licenses/by/4.0/ This content is distributed under the terms of the Creative Commons Attribution 4.0 International license.

### Glycosuria induces UPEC biofilm formation.

**FIG 4:**
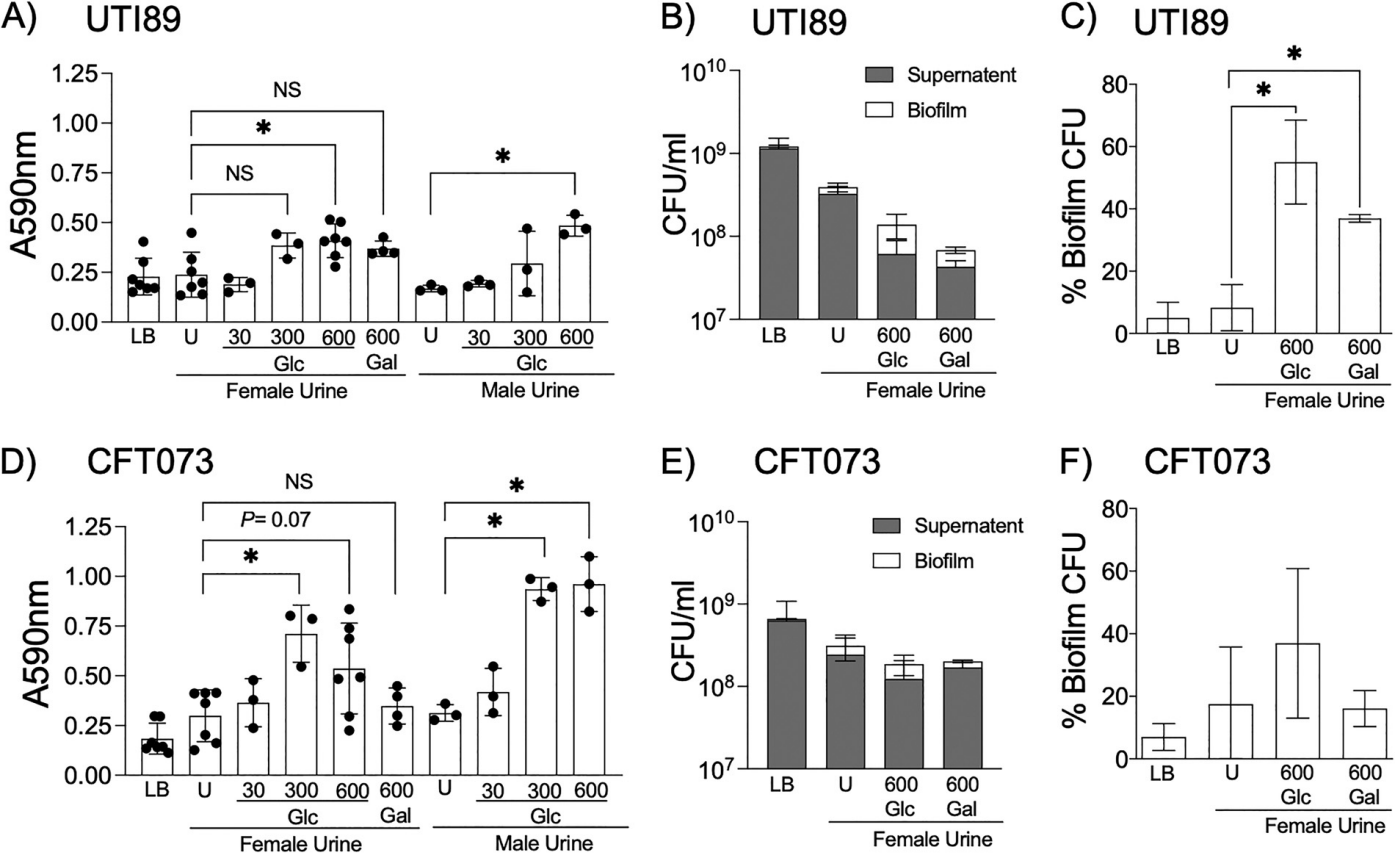
Biofilm formation by UPEC strains in the presence of human urine plain or supplemented with glucose or galactose. Type 1 piliated UTI89 (A, B, and C) and CFT073 (D, E, and F) were cultivated for 24 h in 96-well polystyrene plates at 37°C with 150-rpm shaking in LB or female/male urine either plain or supplemented with various concentrations of glucose or galactose as indicated. (A and D) Biofilms were quantified by staining biomass with crystal violet and presented as scatterplots of at least three biological repeats (each with a minimum of five technical repeats), with histograms indicating averages ± standard deviations. In separate wells, biofilm-bound and planktonic CFU were enumerated by dilution plating and presented as raw CFU values (B and E) or as average percent biofilm-bound CFU from a minimum of two biological replicates ± standard deviations (C and F). Results were compared by ordinary one-way ANOVA followed by Tukey’s multiple-comparison test. *, *P *< 0.05. Nonsignificant *P* values are not shown.

Biofilms are multibacterial communities organized in a three-dimensional space in a matrix made of proteins, polysaccharides, and DNA secreted by biofilm-bound bacteria (35). UPEC produces biofilms on the surface and inside bladder epithelial cells as well as on urinary catheters (36). To determine the effects of glycosuria on biofilm formation, type 1 piliated UPEC was washed in phosphate-buffered saline (PBS) and resuspended in LB, fU, fUG, fUGal, mU, or mUG for biofilm formation in a 96-well, flat-bottom, polystyrene plate. After overnight incubation at 37°C without or with shaking at 150 rpm, biofilms were examined by crystal violet staining and/or by enumerating planktonic (supernatant) and biofilm-bound CFU. Shaking at 150 rpm was meant to mimic shear forces due to gush of urine in the urinary bladder. We observed that increasing glycosuria in either male or female urine augmented biofilm formation by both UTI89 and CFT073 in a dose-dependent manner, as judged by crystal violet staining of biofilm biomass for UTI89 (Fig. 4A) and CFT073 (Fig. 4D). UTI89-fUG also showed significantly higher proportion of biofilm-bound CFU compared to LB-control and UTI89-fU (Fig. 4B and C), while increase in CFT073-fUG CFU numbers was not statistically significant compared to either LB-control or CFT073-U (Fig. 4D and E). Urine alone did not induce biofilm formation in either UTI89 or CFT073 compared to LB controls. Similar results were observed by crystal violet staining of nonshaking biofilms in male/female urine with or without 600 mg/dL glucose (data not shown). Overall, these results suggest that glycosuria augments UPEC biofilms. This contradicts the observations by Hufnagel et al. that exposing UTI89 to 20% (wt/vol) glucose mediates catabolite repression, in turn preventing biofilm formation via cAMP-CRP (25). This apparent contradiction may have resulted from the use of ~33-fold higher glucose concentration by Huffnagel et al. than what was used by us (25). Compared to LB-control, both UTI89-fUGal and CFT073-fUgal showed significantly higher biofilm biomass by crystal violet staining (Fig. 4A and D). CFU enumeration showed increased proportion of biofilm-bound CFU for UTI89-fUGal compared to UTI89-LB or UTI89-fU (Fig. 4C). Overall, these results suggest that UPEC biofilm is augmented by glycosuria as well as by supplementation of urine with galactose, which can be also metabolized by UPEC as a source of carbon (23).

### Glycosuria affects UPEC hemagglutination of guinea pig RBCs.

**FIG 5:**
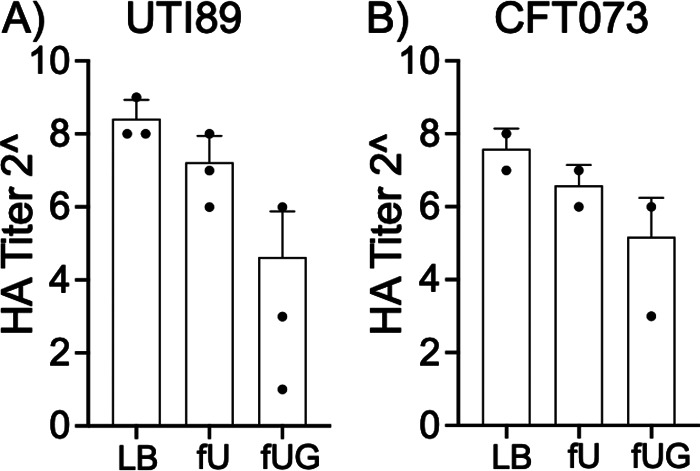
Hemagglutination (HA) titers of UPEC strains exposed to human urine with or without glucose. Type 1 piliated UTI89 (A) and CFT073 (B) preexposed for 2 h to LB, fU, or fUG were washed, adjusted to *A*_540_ = 1.0, diluted 2-fold (from 2^1^ to 2^11^), and mixed with Guinea pig RBCs. After overnight incubation at 4°C, hemagglutination titer was determined visually as the lowest dilution at which RBC button is visible in a V-bottom plate. Average HA titers ± standard deviations from 3 biological replicates for UTI89 and 2 biological replicates for CFT073 are shown. Each biological replicate had three technical replicates.

Adherence of type 1 piliated UPEC to bladder epithelium is the first step in colonization of the urinary tract. Guinea pig hemagglutination (HA) assays were used to examine the assembly of functional type 1 pili on the bacterial surface, where guinea pig red blood cells (RBCs) are mixed with 2-fold serial dilution of UPEC exposed to LB, fU, or fUG either for 2 h (setup 1) or for 24 h (setup 2). HA titer is reported as the lowest bacterial dilution at which UPEC agglutinated guinea pig RBCs. We did not observe significant changes in HA titer in UPEC exposed to fUG or fU for 2 h under setup 1. In contrast, compared to LB-control, HA titers of UTI89-fU and CFT073-fU were ~2-fold decreased, while HA titers for UTI89-fUG and CFT073-fUG were reduced by ~10-fold and 5-fold, respectively, although this reduction was not statistically significant (Fig. 5). In both setups, hemagglutination was inhibited in the presence of soluble mannose.

### Preexposure to glycosuria induces UPEC virulence in a mouse model of ascending UTI.

**FIG 6:**
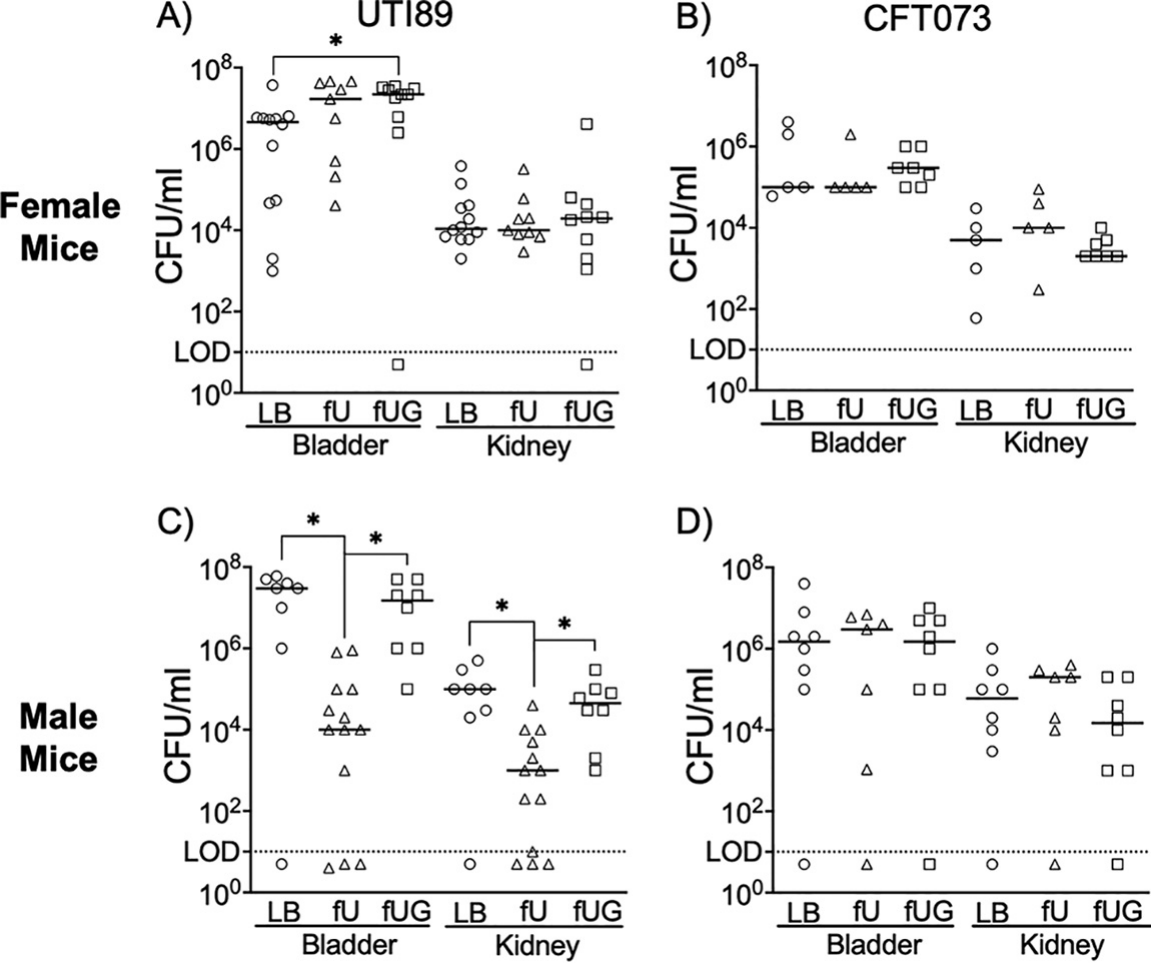
Effects of 2-h-long exposure to human urine with or without glucose on the uropathogenesis of UPEC strains in a mouse model of ascending UTI. Type 1 piliated UTI89 (A and C) and CFT073 (B and D) were exposed for 2 h to LB or female urine with or without 600 mg/dL glucose and washed. C3H females (A and B) and males (C and D) were infected with UPEC from each exposure. At 24 h postinfection, bacterial organ burden in bladder and kidneys was determined by dilution plating the organ homogenates. Scatterplots show number of CFU recovered from individual animals, with median as the measure of central tendency and limit of detection (LOD) as dotted line. The presented data are from a minimum of two biological replicates, each with 3 to 5 mice. Data were analyzed by Mann-Whitney U test (*, *P *< 0.05). Nonsignificant *P* values are not shown.

To examine the effects of glycosuria on UPEC virulence, we experimentally induced ascending UTI in 8-week-old male and female C3H mice by transurethral catheterization of UTI89 or CFT073 preexposed (2 h, 37°C, static) to LB, fU, or fUG. Bladder, kidneys, and spleen homogenates were dilution plated at 24 h postinfection (hpi) to enumerate bacterial organ burden. No bacteria were recovered from the spleen, indicating lack of dissemination via blood. In female mice infected with UTI89-fUG, median bladder CFU numbers were 4.8-fold (*P* = 0.03, two-tailed Mann-Whitney U test) and 1.3-fold (not significant) higher than those from female mice infected with UTI89-LB or UTI89-fU, respectively (Fig. 6A). The median kidney CFU numbers from female mice infected with UTI89-fUG showed <2-fold (not significant) increase compared to female mice infected with UTI89-LB or UTI89-fU, respectively (Fig. 6A). In female mice infected with CFT073-fUG, median bladder CFU numbers were 2-fold (not significant) higher than those from female mice infected with CFT073-LB or CFT073-fU (Fig. 6B). Interestingly, male mice infected with UTI89-fU showed 1,500-fold (*P* < 0.0001) and 3,000-fold (*P* = 0.017) reduction in median bladder bacterial burden and 45-fold (*P* = 0.003) and 100-fold (*P* = 0.003) reduction in median kidney bacterial burden compared to those from male mice infected with UTI89-fUG or UTI89-LB, respectively (Fig. 6C). In contrast, male mice infected with CFT073-fU showed a modest (2- to 4-fold) but statistically insignificant increase in median bladder and kidney CFU numbers compared to their counterparts infected with either CFT073-LB or CFT073-fUG (Fig. 6D). To elucidate sexual dimorphism in the organ burden from UTI89-infected mice (Fig. 6A and C), we infected male C3H mice with UTI89 exposed to male urine with or without 600 mg/dL glucose (mU and mUG). At 24 hpi, male mice infected with UTI89-mU showed 43-fold (*P* = 0.0006) reduction in median bladder bacterial burden and 13-fold (*P* = 0.01) reduction in median kidney bacterial burden compared to those from male mice infected with UTI89-mUG (Fig. S5). Overall, these results indicate that 2-h-long exposure to glycosuria modestly increases UTI89 virulence in female mouse model of ascending UTI while exposure to either male or female urine suppresses UTI89 virulence in male mice.

10.1128/msphere.00004-22.5 FIG S5 Bacterial organ burden at 24 hpi from male C3H mice infected with UTI89 preexposed for 2 h to either plain male urine (mU; open triangle) or to male urine supplemented with 600 mg/dL glucose (open rectangle) (mUG). CFUs recovered from individual mice (two biological replicates) and median (flat line) are shown. *, *P *< 0.05, Mann-Whitney U test. Download FIG S5, PDF file, 0.03 MB. Copyright © 2022 Islam et al. 2022 Islam et al. https://creativecommons.org/licenses/by/4.0/ This content is distributed under the terms of the Creative Commons Attribution 4.0 International license.

## DISCUSSION

A nondiabetic urinary tract is a moderately oxygenated environment containing urine made of low concentrations of amino acids and short peptides and trace amounts of transition metals, including iron (28, 29, 33). To successfully colonize nondiabetic urinary tract, UPEC must import and catabolize amino acids and short peptides via TCA cycle and gluconeogenesis. Based on the comparative transcriptomic analysis of UPEC isolated from human patients with uncomplicated UTI, Sintsova et al. have proposed that during UTI, UPEC shuts down metabolic machinery and diverts all available resources to replication and translational gene expression (37). In the presence of glycosuria, however, UPEC is expected to utilize glucose as a ready carbon source through glycolysis. The primary objective of this project was to assess whether such a shift toward glycolytic metabolism in the presence of glycosuria favors the fitness of UPEC in the urinary tract. For this, we compared UPEC physiology in either plain human urine or human urine supplemented with glucose.

The importance of peptide transport across bacterial membrane, gluconeogenesis, and TCA cycle in uropathogenesis of UPEC has been established in mouse models of ascending UTI, with UPEC deletion mutants targeting key enzymes of these pathways in the mice infected with WT UPEC (38–41). We observed that transferring UTI89 from nutrient-rich LB to either plain human urine (UTI89-fU) or to glycosuria (UTI89-fUG) rapidly upregulates expression of genes encoding transporters of amino acids serine, arginine, and histidine, branched-chain amino acids (leucine, isoleucine, and valine), and dipeptides, suggesting that UTI89 continues to rely on utilization of amino acids. This concurs with (i) the observations from examination of transcriptome of UPEC isolated from the urine of humans with uncomplicated UTI or from the mouse urinary tract at 48 hpi (33, 42) and (ii) with previous research that UPEC mutants deficient in peptide transport (Δ*dppA*) are impaired in colonizing murine bladder and kidney tissues, while UPEC auxotrophs for arginine, serine, or glycine are not defective in a mouse model of ascending UTI, indicating that UPEC can import these amino acids from the urinary tract and metabolize them (39). Whether long-term access to glucose in urinary tract with glycosuria diverts UPEC from amino acid catabolism to glucose utilization remains to be determined. This idea is supported by RNA-seq observations that the expression of genes encoding enzymes from central carbon metabolism pathways, such as glycolysis/gluconeogenesis and TCA cycle, were significantly altered by 2-h exposure to fUG.

Catalytic activity and structural stability of almost half of the enzymes from both the pathogen and its eukaryotic host are dependent on acquisition of transition metals such as iron, manganese, nickel, cobalt, zinc, and copper (43). These transition metals are sequestered in various host proteins, such as heme, ferritin, transferrin, and ceruloplasmin, because of the toxicity associated with their presence in free ion form. Transition metal sequestration also gives the host a competitive advantage over pathogens. In response to iron limitation stress, E. coli downregulates high-ATP-yielding pathways, such as TCA cycle, whose functioning depends on a number of enzymes containing iron-sulfur clusters (44). In the iron-limiting urinary tract, UPEC limits investment of iron by downregulating oxidative TCA cycle enzyme FumA (class I fumarate hydratase), which requires iron for its activity, while inducing expression of FumC fumarate hydratase, which does not require iron for activity (41, 45). FumC is critical for uropathogenesis of UPEC, as a Δ*fumC* mutant has been shown to be defective in mouse model of ascending UTI (39–41). Similar to these observations, we observed that, compared to UTI89-LB, UTI89-fU had significant upregulation of *fumC*, while UTI89-fUG did not exhibit significant change in *fumC* expression. Moreover, RNA-seq analysis showed that exposure to glycosuria rapidly and uniformly activated genes encoding free metal ion uptake systems (*sitABCD*), siderophore-mediated metal ion uptake systems (*iro*-salmochelin and *ybt*-yersiniabactin), and importers of host protein-conjugated metal ions (Chu). Based on these observations, we hypothesize that glycosuria-mediated upregulation of iron uptake systems restores the activity of proteins whose activity is dependent on iron, such as key TCA cycle enzymes like FumC and the components of electron transport chains. Notably, the divergent metabolic paths taken by UTI89-fUG and UTI89-fU do not appear to directly affect UPEC growth based on the observations that UPEC grew rapidly in human urine irrespective of glucose supplementation.

Next, we addressed whether a metabolic switch toward glycolysis provides a direct fitness advantage to UTI89 in diabetic urinary tract. We inoculated UTI89 and CFT073, exposed for 2 h to LB, U, or UG, into male and female C3H mice via transurethral catheterization. Results show that compared to UTI89-LB control, UTI89-fUG has modest but statistically significant fitness advantage in the colonization of female mouse bladder. A similar but statistically insignificant trend was observed for CFT073. Whether long-term exposure to glycosuria would augment UPEC virulence even further must be examined in the future.

The significant differences observed between UTI89 organ burdens in male and female mice were particularly striking. Compared to their counterparts infected with UTI89-LB, UTI89-fU-infected female mice showed a modest increase in bladder CFU numbers, while UTI89-fU-infected male mice showed a significant reduction in both bladder and kidney CFU numbers. We sought to define the molecular effector(s) underlying these differences. Exposure to either male or female urine did not change gene expression profiles (Fig. 4) or biofilm formation (Fig. 5) in either UTI89 or CFT073. Thus, neither biofilm formation nor the changes in the expression of specific genes examined using qRT-PCR can be used to explain sexual dimorphism observed in mouse experiments. Moreover, despite similarities in the observed effects of urine with or without glucose on the physiology of CFT073 and UTI89, male mice infected with CFT073-fU did not exhibit reduction in bladder/kidney bacterial burden compared to males infected with CFT073-LB, indicating UTI89-specific nature of this phenotype. We further explored differences observed in the organ bacterial burdens of male and female mice infected with UTI89-fU by infecting male C3H mice with UTI89 exposed to male urine with or without 600 mg/dL glucose (Fig. S6). However, compared to male mice infected with UTI89-mUG, UTI89-mU-infected male mice showed a reduction in bladder and kidney CFU similar to that observed in male mice infected with UTI89-fU. Future RNA-seq experiments comparing transcriptomes of UPEC isolated from the urinary tracts of male and female mice will be useful in identifying whether male urine specifically affects the expression of gene(s) critical to urinary pathogenesis. The organ burden disparity between CFT073-fU- and UTI89-fU-infected male mice can be attributed to the considerable differences between CFT073 and UTI89 in their repertoire of virulence factors and their expression levels in the urinary tract or to the strain-specific immune responses.

Our RNA-seq results showed that 2-h exposure to fU or fUG significantly upregulates *fimE* (UTI89_C5010) and significantly downregulates *fimB* (UTI89_C5009) without affecting the expression of polycistronic *fim* operon. FimE and FimB are transacting, DNA-binding proteins, of which FimE binding inverts *fimS* to phase-OFF orientation and prevents transcription of *fim* operon proteins involved in type 1 pilus assembly (46). According to our results, both UTI89 and CFT073 exhibited reduced HA titer when cultivated for 48 h in the presence of female urine, suggesting a corresponding reduction in assembly and/or function of type 1 pili; HA titer was further reduced in UPEC strains exposed to glycosuria. However, 2-h exposure to fU or fUG did not affect hemagglutination activity in either of the two strains. Human urine not only inhibits the expression of *fim* operon by inducing *fimS* phase-OFF orientation in UTI89 cultivated under type 1 piliation conditions (static, 37°C, two 24-h cultures; see Materials and Methods) but also suppresses the function of FimH adhesive tip (47). In contrast, the growth of UPEC in glucose-supplemented minimal medium reduces levels of cAMP-CRP, in turn increasing FimB-mediated inversion of *fimS* to phase-ON orientation and promoting type 1 piliation (48). RNA-seq analysis of UTI89 exposed for 2 h to LB, fU, or fUG showed that transcript levels of *cyaA* (encodes adenylyl cyclase enzyme catalyzing cAMP synthesis, UTI89_4365) and *crp* (encodes CRP, UTI89_C3860) were unaffected in either UTI89-fU versus UTI89-LB or in UTI89-fUG versus UTI89-fU comparisons. Further experiments are needed to elucidate the effects of 48-h-long cultivation in the presence of glycosuria on the expression of *fim* operon regulators (*fimE* and *fimB*) and/or cAMP-CRP system (*cyaA* and *crp*).

Cultivation of UPEC strains in glycosuria augmented biofilms as compared to that of UPEC in LB-control or plain urine. Similar biofilm augmentation was observed with male and female urine. Addition of equal amounts of galactose to urine also significantly increased biofilm formation compared to that of LB-control. Thus, biofilm augmentation in UTI89-UG and CFT073-UG appears to be modulated at least partially via changes in the osmolarity of urine. Albeit with two important caveats that this experimental setup: (i) does not consider the confounding effects of UPEC central carbon metabolism being diverted toward galactose utilization (23), and (ii) does not determine the effects of ionic osmolytes on UPEC gene expression or biofilm formation.

Glucose-mediated catabolite repression is known to inhibit biofilm formation via reduction in cAMP-CRP, an inducer of biosynthesis of surface adhesive type 1 pili, curli fibers, flagella, and K1 capsule (24, 25). Compared to UTI89-fU, UTI89-fUG showed significant downregulation of *csgD* (UTI89_C1161) and *flhCD* (UTI89_C2094/2095), positive regulators of curli fiber and flagellar gene expression, respectively, while K1 capsule-encoding operon (UTI89_C3362 to UTI89_C3367) was significantly downregulated in UTI89-fUG compared to UTI89-LB. As our transcriptome data show transcript levels following 2-h-long exposure to LB, fU, or fUG, it does not truly reflect gene expression changes in UPEC strains cultivated overnight in the presence of LB or urine with or without glucose for biofilm formation. Overall, to further elucidate implications of these observations to UTI pathogenesis, future experiments examining expression of surface adhesins in UPEC from diabetic and nondiabetic murine urinary tracts are warranted.

In summary, results presented in this report provide significant insights into changes in UPEC physiology mediated by human urine with or without glucose. An important shortcoming, that our experimental design may not truly reflect changes in UPEC physiology during long-term infection of the urinary tract of a diabetic host, must be addressed by future experiments analyzing the physiology (virulence and transcriptomes) of UPEC isolated from the urinary tracts (bladder and kidney tissues) of diabetic and nondiabetic mice at various time points after experimental induction of ascending urinary tract infection.

## MATERIALS AND METHODS

### Human urine collection.

As approved by institutional review board at UL Lafayette, urine was collected from healthy male and female volunteers (18 to 45 years of age) who had not suffered from UTI and/or been treated with antibiotics in a month prior to urine collection. Urine was sterilized using 0.22-μm syringe filter and stored at −80°C in 1.5-mL or 15-mL aliquots. At the time of the experiments, urine aliquots from at least 3 separate male or female donors were pooled. Pooled urine (from both male and female donors) had pH of 7.0 and specific gravity of 1.0.

### Bacterial strains and growth conditions.

As model UPEC strains, we used UTI89, a bladder isolate from an acute cystitis patient, and CFT073, a blood and urine isolate from a pyelonephritis patient, sourced from the Thanassi lab strain collection (Stony Brook University) (20, 21). Prior to experimentation, UPEC strains were cultured at 37°C in LB in a static culture for 24 h, followed by 1:100 subculture into fresh LB and incubation at 37°C, static for an additional 24 h to induce type 1 piliation (22). In this report, UPEC cultures prepared in this manner are described as type 1 piliated.

Type 1 piliated UTI89 and CFT073 were pelleted, washed in sterile PBS, and then resuspended in LB or in pooled human urine, either plain or supplemented with 30, 300, or 600 mg/dL glucose or 600 mg/dL galactose. UPEC cultures were maintained at 37°C, static for various lengths of time as indicated below. fU refers to plain female urine, mU to plain male urine, fUG to female urine plus 600 mg/dL glucose, mUG to male urine plus 600 mg/dL glucose, and fUGal to female urine plus 600 mg/dL galactose.

### Growth curve.

UPEC strains CFT073 and UTI89 were maintained at 37°C in LB, fU, or fUG. CFU were enumerated by dilution plating at 0, 1, 2, 4, 6, 8 and 24 h. The doubling time (DT) was calculated as

$$\text{DT}=\frac{{\text{Duration(log}}_{2}\text{)}}{\text{log}\left(\frac{\text{CFU}}{\text{ml}}\text{ at 4 h}\right)\text{ – log}(\frac{\text{CFU}}{\text{ml}}\text{ at 2 h})}$$

### *In vitro* biofilm assay.

Type 1 piliated UTI89 or CFT073 was pelleted, washed, and resuspended in LB or in mU or fU supplemented with 0, 30, 300, or 600 mg/dL glucose or 600 mg/dL galactose. Next, 100 μL of each culture was transferred to the wells of a 96-well sterile polystyrene plate and incubated overnight at 37°C with or without shaking at 150 rpm. We have previously described detailed protocols for biofilm visualization by crystal violet staining or CFU enumeration (49, 50). In short, after 3 rigorous washes with sterile PBS, biofilm-bound bacteria were scraped off with a pipette tip and enumerated by dilution plating. Planktonic CFU numbers were determined by dilution plating the supernatant collected prior to washing. In separate experiments, rigorously washed biofilms were dried, baked at 60°C, and stained with crystal violet. Biofilm biomass is directly proportional to retained crystal violet, which was extracted in ethanol-methanol mix, and color intensity of the extract was measured at 590-nm absorbance.

### Hemagglutination of guinea pig RBCs.

Effects of glycosuria on type 1 pilus formation by UPEC was examined by mannose-sensitive agglutination of guinea pig erythrocytes (51). In setup 1, type 1 piliated UTI89 and CFT073 were exposed to LB, fU, or fUG for 2 h at 37°C under static conditions. In setup 2, UTI89 or CFT073 was cultivated in LB, fU, or fUG for 48 h at 37°C under static conditions. After exposure, bacterial pellets (4,080 × *g*, 10 min, 4°C) were resuspended in sterile PBS to *A*_540_ = 1.0 and maintained on ice. Citrated guinea pig blood (Colorado Serum Company) was centrifuged at 230 × *g*, 4°C. RBC pellet was washed in cold sterile PBS until supernatant was clear (no red color). RBCs were then set to *A*_640_ = 1.9. For hemagglutination assay, 25 μL bacteria was 2-fold diluted in sterile PBS 12 times (from undiluted and 2^0^ to 2^11^) in three technical replicates in a V-bottomed plate. A volume of 25 μL RBCs then was added to each well, mixed by tapping plate sides, covered with plate sealer, and incubated overnight at 4°C. To ensure that hemagglutination was caused by type 1 pili, in separate wells 2% mannose (wt/vol in sterile PBS) was added prior to addition of guinea pig RBCs. Mannose competitively inhibits type 1 pilus binding to guinea pig RBCs. For determining HA titer, plates were visually examined for the highest dilution showing RBCs as a red button at the bottom of the V-shaped well. Control wells contained LB, fU, or fUG without bacteria.

### RNA extraction, reverse transcription, and qRT-PCR.

Type 1 piliated UPEC strains were pelleted and exposed for 2 h at 37°C, static, to LB, fU, fUG, or fUGal or in separate experiments to LB, mU, or mUG. Bacterial RNA was extracted using Ambion Ribopure kit (Thermo Fisher Scientific) and treated with DNase. The quality (*A*_260_/*A*_280_) and quantity of extracted RNA was determined by NanoDrop (Synergy HTX multi-mode microplate reader; BioTek). Next, 1 μg total RNA was reverse transcribed using the Applied Biosystems high-capacity cDNA reverse transcription kit (Thermo Fisher Scientific). qRT-PCR was carried out using Power SYBR green master mix (Applied Biosystems) in StepOne Plus thermal cycler (Applied Biosystems). List of primers used for qRT-PCR is shown in Table 5. Relative quantification (RQ) values were calculated by comparative threshold cycle (ΔΔ*C_T_*) algorithm (34) with normalization to 16S rRNA. RQ fold differences over transcript levels from LB-control are presented as averages from at least two biological replicates (each with three technical repeats) ± standard deviations.

### Mouse model of ascending UTI.

To examine changes in the virulence of UPEC mediated by exposure to glycosuria, male and female C3H mice were infected via transurethral catheterization with 10^7^ CFU of UTI89 or CFT073 preexposed to LB, fU, or fUG (2 h, 37°C, static) resuspended in 50 μL sterile PBS. The protocol for transurethral catheterization of uropathogens to induce ascending UTI (approved by UL Lafayette IACUC) was previously described in detail (22, 52). At 24 hpi, bacterial burden in bladder, kidneys, and spleen was determined by diluting organ homogenates in sterile PBS and plating the dilutions on sterile lysogeny agar (LB agar) plates. The mouse experiments were repeated a minimum of two times (biological replicates), each with 3 to 5 mice. Organ burden data are presented as scatterplots with median as central tendency.

### Statistical analysis.

Data from multiple biological replicates for each experiment were pooled. Graphing and statistical analyses were done using GraphPad Prism 9.2.0. qRT-PCR, biofilm, and hemagglutination results are expressed as averages ± standard deviations from two or more biological replicates, each with two or more technical replicates. These data were compared by one-way ANOVA followed by Tukey’s multiple comparisons or by Student's *t* test. The organ burden data were compared using Mann-Whitney U statistic. The difference between groups is considered significant at a *P* value of ≤0.05.

### RNA-seq and data analysis.

Total RNA extracted from UTI89 preexposed to LB, fU, or fUG (2 h, 37°C, static, three biological replicates) was examined for RNA quality, quantity, and DNA contamination using Qubit (Thermo Fisher Scientific) and Tapestation (Agilent). All library construction and initial analysis of differential expression was done by GENEWIZ (NJ, USA). Library construction included DNase treatment (TURBO DNase; ThermoFisher Scientific) and rRNA depletion (QIAseq FastSelect; Qiagen) followed by RNA fragmentation and random priming. cDNA synthesis (NEBNext Ultra II; New England Biolabs) was followed by end repair, 5′ phosphorylation, and dA tailing. Libraries were sequenced on a partial lane of Illumina HiSeq 4000 with 150-bp PE sequencing. Quality of sequence data was assessed using FastQC. All reads were quality filtered and trimmed using Trimmomatic v 0.36 with default settings (53). Reads were mapped to Escherichia coli O18:K1:H7 UTI89 (UPEC) genome using bowtie v 2.2.6 (54), and hit counts for individual genes were generated using the featurecounts command in the subreads package v 1.5.2 (55). Genes with <10 reads were dropped from the analysis for differential expression. Differential expression for each gene was assessed using Wald tests implemented in DESeq2 (56). Genes with an adjusted *P* value (*P*adj) of <0.05 and absolute log_2_ fold change (log_2_FC) of >1 were categorized as differentially expressed genes (DEG).

KEGG pathway enrichment analyses were conducted using the KEGGREST Bioconductor package v1.30.1 (57) and a custom script (available upon request). KEGGREST was used to download lists of pathways and genes within pathways from the KEGG website for Escherichia coli O18:K1:H7 UTI89 (UPEC) (organism code *eci*). We assessed whether DEGs are overrepresented in certain pathways using a Wilcoxon rank-sum test to determine whether adjusted *P* values for differential expression of genes within a focal pathway are less than the adjusted *P* values for differential expression of genes that are not within the pathway.

### Data availability.

The raw Illumina reads have been deposited at NCBI’s BioProject database under accession no. PRJNA786257.
