# Supplementary material for: Glycosuria Alters Uropathogenic Escherichia coli Global Gene Expression and Virulence
Source: mSphere. 2022 Apr 28;7(3):e00004-22. doi: 10.1128/msphere.00004-22 (PMC9241551; doi:10.1128/msphere.00004-22)

Fig S1. Principal-component analysis and Euclidean distance between samples for UTI89-LB versus UTI89-fU (A and B), UTI89-LB versus UTI89-fUG (C and D), and UTI89-fU versus UTI89-fUG (E and F).

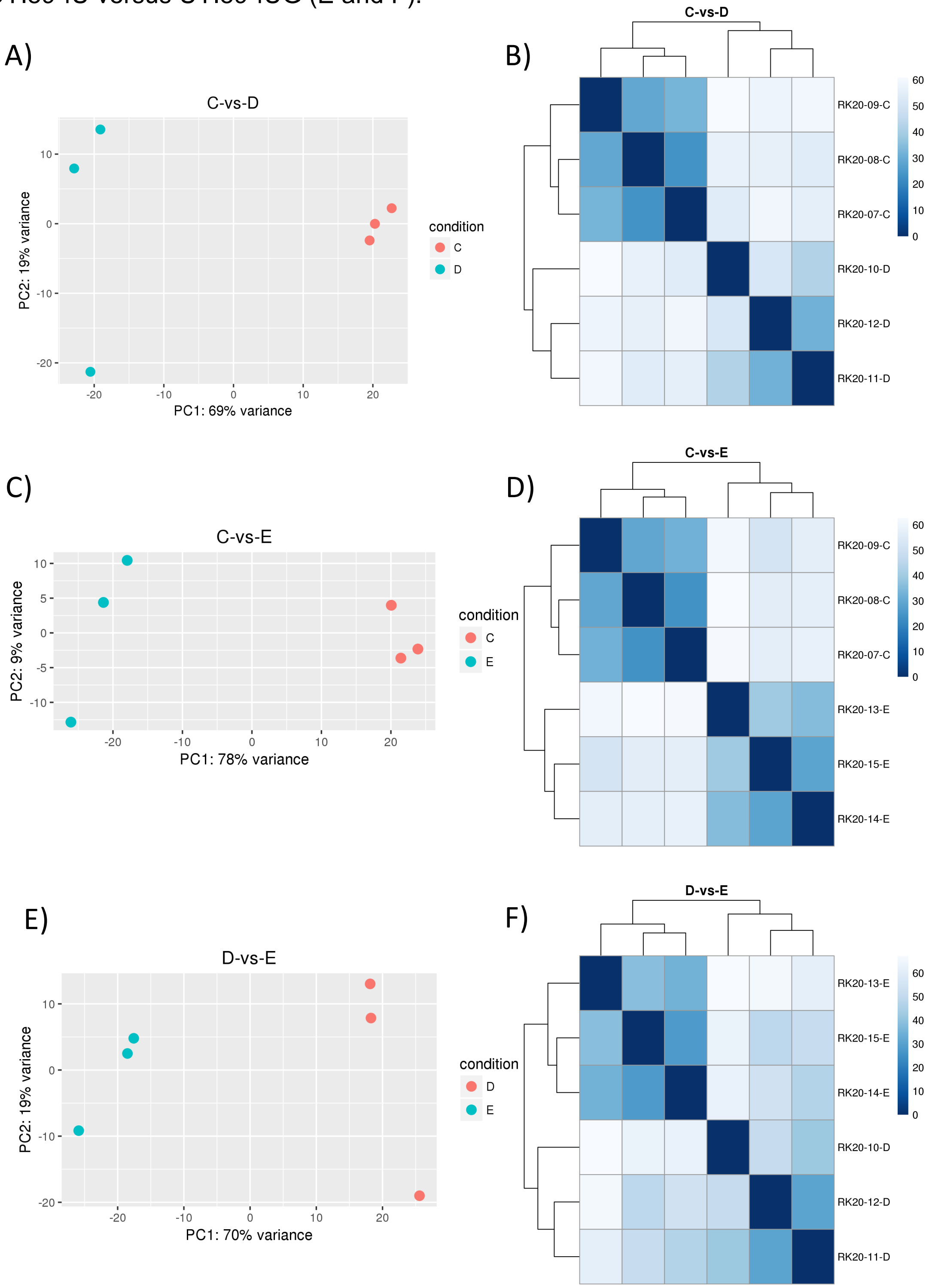

Supplement: FIG S1 [file msphere.00004-22-s0002.pdf]
