# Supplementary material for: Glycosuria Alters Uropathogenic Escherichia coli Global Gene Expression and Virulence
Source: mSphere. 2022 Apr 28;7(3):e00004-22. doi: 10.1128/msphere.00004-22 (PMC9241551; doi:10.1128/msphere.00004-22)

S2. Correlation between qRTPCR and RNASeq results for (A) UTI89-fU compared to UTI89-LB and (B) UTI-89-fUG compared to UTI89-LB

A) UTI89-fU compared to UTI89-LB

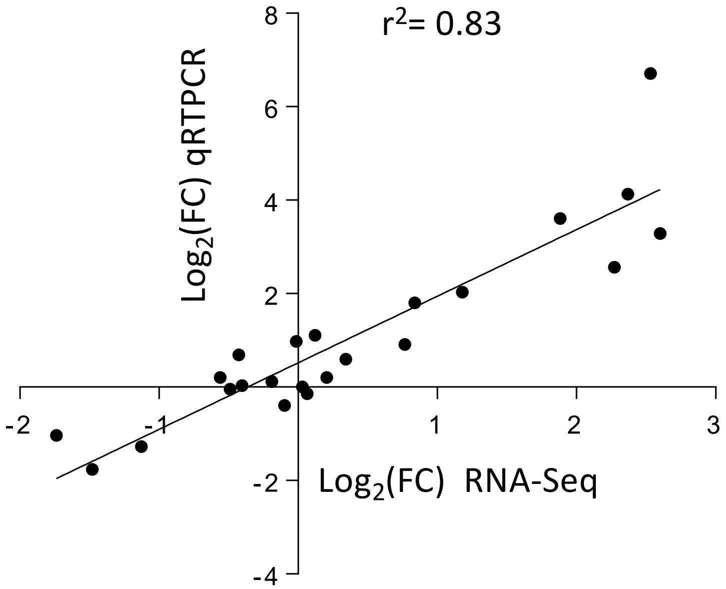

B) UTI89-fUG compared to UTI89-LB

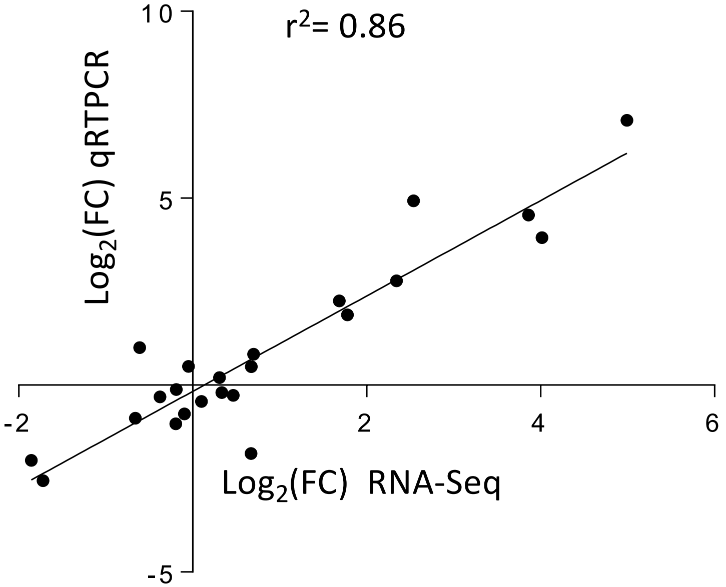

Supplement: FIG S2 [file msphere.00004-22-s0003.pdf]
