# Supplementary material for: Glycosuria Alters Uropathogenic Escherichia coli Global Gene Expression and Virulence
Source: mSphere. 2022 Apr 28;7(3):e00004-22. doi: 10.1128/msphere.00004-22 (PMC9241551; doi:10.1128/msphere.00004-22)

S3. KEGG pathways significantly enriched for DEG at *P*<0.01 are shown for three comparisons (A) UTI89-LB versus UTI89-fU, (B) UTI89-fUG versus UTI89-LB, and (C) UTI89-fUG versus UTI89-fU. The total number of genes in each KEGG pathway is shown next to the histogram.

A) UTI89-LB Vs UTI89-fU

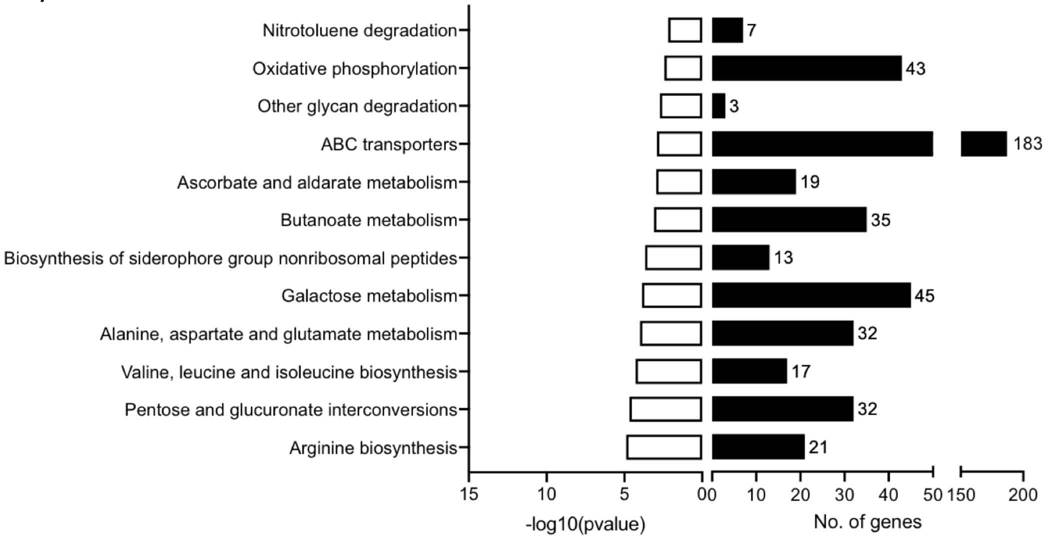

B) UTI89-UG Vs UTI89-fU

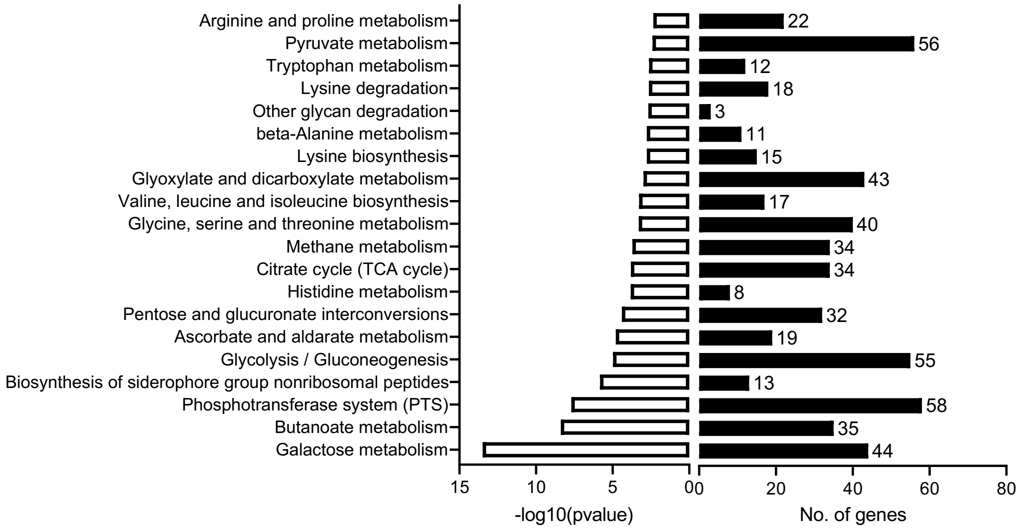

C) UTI89-LB Vs UTI89-fUG

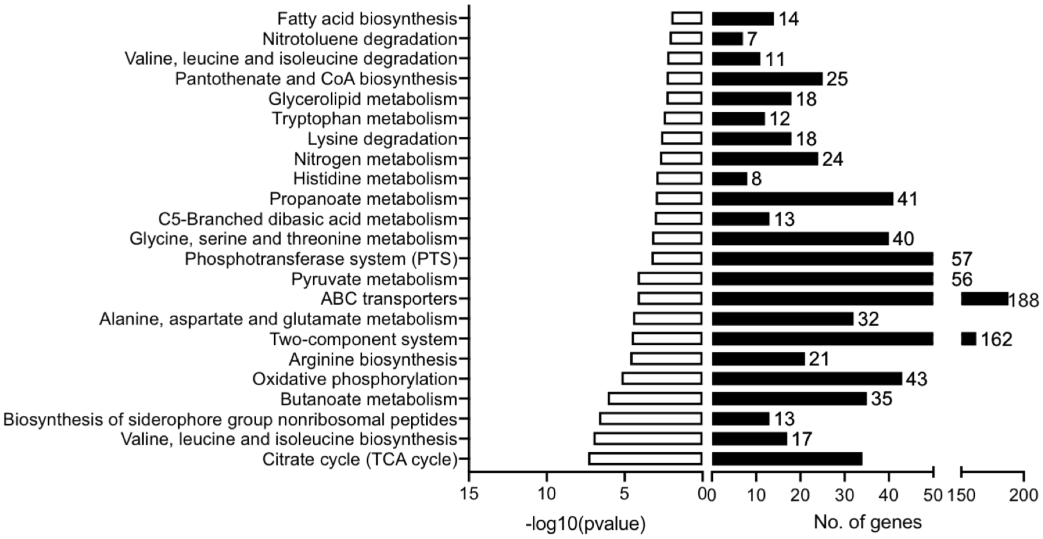

Supplement: FIG S3 [file msphere.00004-22-s0004.pdf]
