# Supplementary material for: Glycosuria Alters Uropathogenic Escherichia coli Global Gene Expression and Virulence
Source: mSphere. 2022 Apr 28;7(3):e00004-22. doi: 10.1128/msphere.00004-22 (PMC9241551; doi:10.1128/msphere.00004-22)

S4. qRT-PCR analysis of expression of metabolic and virulence genes. RQ fold differences for specific mRNA transcript levels for UTI89-fU or UTI89-fUG over transcript levels from LB-control are presented as average of at least two biological replicates (each with three technical repeats) ± standard deviation

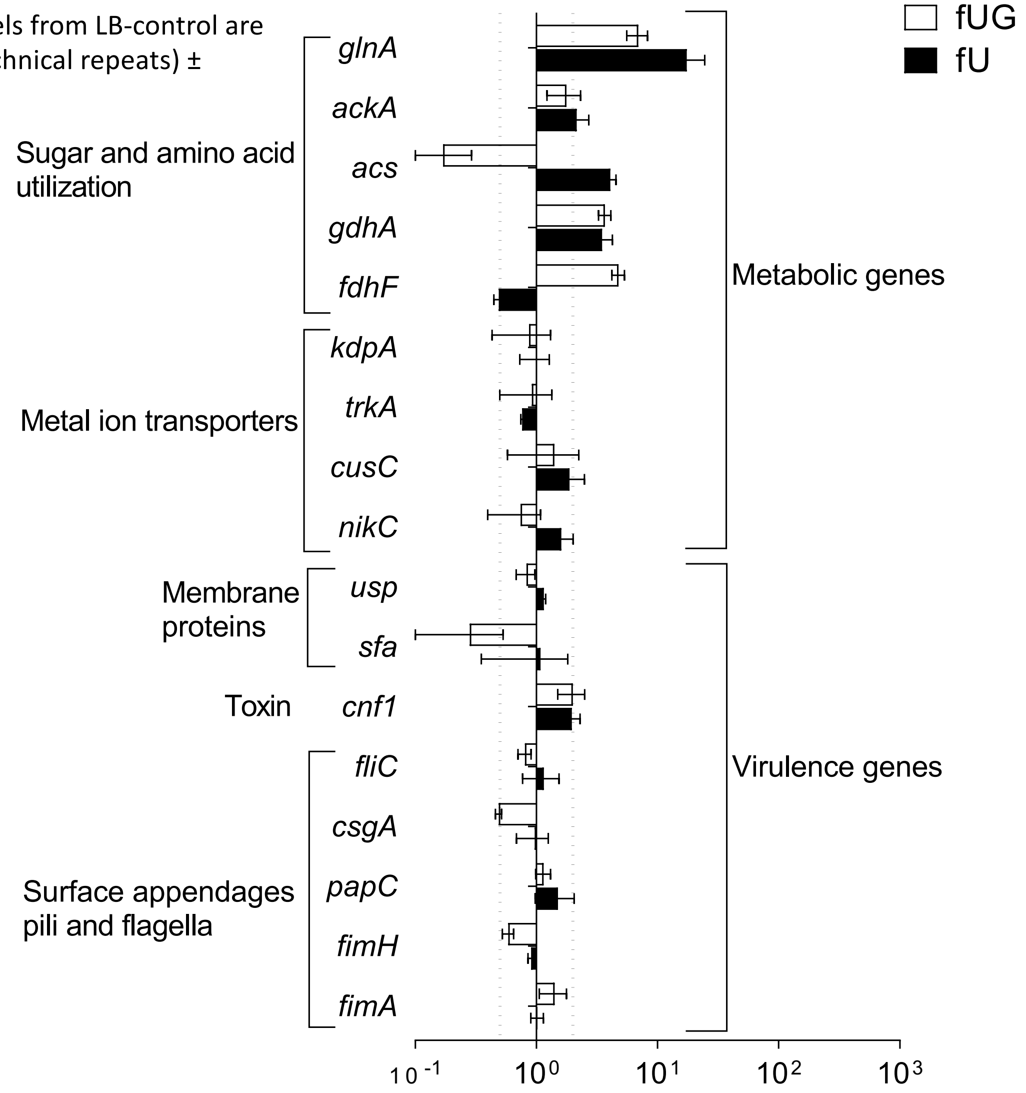

Supplement: FIG S4 [file msphere.00004-22-s0005.pdf]
