# Supplementary material for: Glycosuria Alters Uropathogenic Escherichia coli Global Gene Expression and Virulence
Source: mSphere. 2022 Apr 28;7(3):e00004-22. doi: 10.1128/msphere.00004-22 (PMC9241551; doi:10.1128/msphere.00004-22)

S5. Bacterial organ burden at 24 hpi from male C3H mice infected with UTI89 pre-exposed for 2h to either plain male urine (mU, open triangle) or to male urine supplemented with 600 mg/dl glucose (open rectangle) (mUG). CFUs recovered from individual mice (two biological replicates) and median (flat line) are shown. *, *P*<0.05, Mann-Whitney U test.

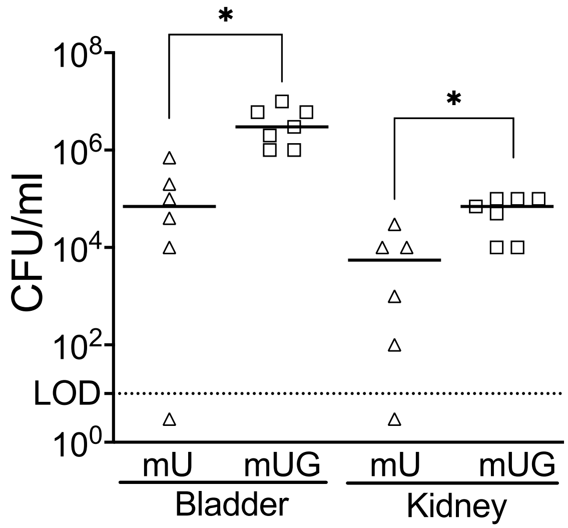

Supplement: FIG S5 [file msphere.00004-22-s0006.pdf]
